# HNF4A guides the MLL4 complex to establish and maintain H3K4me1 at gene regulatory elements

**DOI:** 10.1038/s42003-024-05835-0

**Published:** 2024-01-31

**Authors:** Avinash Thakur, Kwangjin Park, Rebecca Cullum, Bettina M. Fuglerud, Mina Khoshnoodi, Sibyl Drissler, Tabea L. Stephan, Jeremy Lotto, Donghwan Kim, Frank J. Gonzalez, Pamela A. Hoodless

**Affiliations:** 1 Terry Fox Laboratory, BC Cancer, Vancouver, V5Z 1L3 Canada; 2 Department of Medical Genetics, University of British Columbia, Vancouver, V6T 1Z4 Canada; 3 Cell and Developmental Biology Program, University of British Columbia, Vancouver, V6T 1Z4 Canada; 4 Center of Cancer Research, National Cancer Institute, Bethesda, 2089 USA; 5 School of Biomedical Engineering, University of British Columbia, Vancouver, V6T 1Z4 Canada

**Keywords:** Epigenetics, Cell biology, Transcriptional regulatory elements

## Abstract

Hepatocyte nuclear factor 4A (HNF4A/NR2a1), a transcriptional regulator of hepatocyte identity, controls genes that are crucial for liver functions, primarily through binding to enhancers. In mammalian cells, active and primed enhancers are marked by monomethylation of histone 3 (H3) at lysine 4 (K4) (H3K4me1) in a cell type-specific manner. How this modification is established and maintained at enhancers in connection with transcription factors (TFs) remains unknown. Using analysis of genome-wide histone modifications, TF binding, chromatin accessibility and gene expression, we show that HNF4A is essential for an active chromatin state. Using HNF4A loss and gain of function experiments in vivo and in cell lines in vitro, we show that HNF4A affects H3K4me1, H3K27ac and chromatin accessibility, highlighting its contribution to the establishment and maintenance of a transcriptionally permissive epigenetic state. Mechanistically, HNF4A interacts with the mixed-lineage leukaemia 4 (MLL4) complex facilitating recruitment to HNF4A-bound regions. Our findings indicate that HNF4A enriches H3K4me1, H3K27ac and establishes chromatin opening at transcriptional regulatory regions.

## Abstract (web summary)

An interaction between hepatocyte nuclear factor 4A (HNF4A) and the MLL4 complex is important for histone modifications at cis regulatory regions to alter gene expression in vivo and in vitro.

## Introduction

Hepatocyte nuclear factor 4A (HNF4A, aka NR2a1) belongs to the nuclear receptor family of transcription factors (TF)^1^ and functions to control cell identity^2,3^ in hepatocytes^4,5^, renal proximal tubular cells^6^ and enterocytes^7,8^. In adult mouse liver, HNF4A inhibits hepatocyte proliferation^9,10^, and during liver regeneration, it promotes reacquisition of the differentiated phenotype^11^. Induced HNF4A expression, in combination with other TFs, converts fibroblasts to induced hepatocyte-like cells^12–14^. A FOXA factor and HNF4A are sufficient to drive the liver-specific gene expression program, suggesting that these two factors are key in initiating changes^12^. In liver disorders, HNF4A attenuates liver fibrosis and cirrhosis^15^ while its loss induces dedifferentiation, upregulation of genes involved in cancer^10^ and growth of hepatocellular carcinoma (HCC)^16,17^. Overall, HNF4A plays a major role in the balance between hepatic cell proliferation, differentiation and maintenance of cell identity.

In liver, HNF4A is also a critical regulator of epithelial to mesenchyme transition or the reverse (EMT/MET)^18^ as silencing in mature hepatocytes results in upregulation of *Snai2/Slug* and activation of the mesenchymal gene program leading to EMT^19^. Conversely, forced overexpression of HNF4A can induce MET in hepatoma cells^17^ and blocks EMT and HCC initiation in rat models, suggesting its role as a tumour suppressor in liver^16^. Although, HNF4A drives a cell-type-specific gene expression program, the molecular mechanism through which HNF4A exerts its functions is not well understood.

Gene expression is regulated by diverse cis-regulatory elements, known as promoters, enhancers, insulators and silencers, which recruit proteins to act on chromatin. Among these, enhancers play a key role in controlling the expression of genes that establish cell identity and function as a binding platform for TFs. In the case of HNF4A, the majority of DNA-binding sites are found at enhancers. The ability of a TF to activate gene expression depends on the recruitment of co-activator proteins to these elements^20,21^. These co-activators may not bind DNA directly and instead function as either histone modifiers (for example, the histone acetyltransferase p300)^22^, and/or chromatin remodellers^23^ that act by establishing enhancer-promoter interactions to activate or repress gene expression^24–26^.

Histones can be modified with additions of methyl-, acetyl-, phospho- and ubiquityl groups, among others, to change the chromatin conformation and accessibility of DNA^27,28^. Enhancers are marked with a unique set of epigenetic signatures^29–31^ with methylation of histone 3 at lysine 4 (H3K4me) as a key histone modification^32^. Monomethylation on H3K4 (H3K4me1) in combination with acetylation of H3 at K27 (H3K27ac) designates the active enhancer state^33^ while H3K4me1 alone, or with trimethylation of H3K27 (H3K27me3), defines a primed or repressed enhancer, respectively^30^. Of note, H3K4me1 specifically is involved in nucleosome positioning at enhancers and loss of H3K4me1 reduces H3K27ac levels. In contrast, H3K27ac has a stronger influence on enhancer transcription^34^. Mixed-lineage leukaemia 4 (MLL4, also known as KMT2D), alongside the closely related MLL3 (KMT2C), is a histone methyltransferase that establishes H3K4me1/2 at enhancers in mammalian cells^35–37^. MLL4 exists in a multi-protein complex containing the core components WDR5, ASH2L, RBBP5 and DPY30^38,39^ and other complex-specific proteins, such as PAGR1 (previously known as PA1), KDM6A (UTX), NCOA6 and PAXIP1 (PTIP)^37,40^.

Loss of function mutations in MLL4 reduce global H3K4me1 levels, cause abnormal gene expression, and lead to developmental defects or cancer^37^. Interestingly, while MLL4 is required for cell fate transition, not all enhancers are primed prior to differentiation and MLL4 is not required for the maintenance of cell identity^41^. MLL4 binding at enhancers has been shown to coincide with the binding of master regulators^42–44^. While some studies show that signalling-dependent and cell type-specific TFs are capable of recruiting MLL4 to establish H3K4me1 at enhancers^44–47^, whether HNF4A has this function is not known.

While we previously showed that HNF4A is required for active histone and DNA signatures at enhancers^48^, its involvement in establishing the primed state that is laid down prior to activation was not examined. HNF4A bound regions (HBRs) are flanked by nucleosomes marked with H3K4me1^48–50^, leading us to question whether HNF4A contributes to the establishment and maintenance of H3K4me1 at HBRs. In this study, we used a liver-specific, HNF4A conditional knockout (cKO) mouse model and cell line expression models to analyze how HNF4A affects H3K4me1, H3K27ac and chromatin accessibility. Importantly, our data indicate that HNF4A is required for the maintenance of H3K4me1, H3K27ac and the open chromatin state at HBRs in vivo, and can establish these epigenetic features in unmarked chromatin when ectopically expressed in cell lines. Furthermore, although FOXA factors act as pioneer factors in hepatocytes^51,52^, we show that FOXA factors are not always required for accessibility at HBRs. Finally, we demonstrate that HNF4A interacts with the MLL4 histone methyltransferase complex and recruits it to HBRs. Overall, our data support a role for HNF4A in facilitating the establishment of H3K4me1 to initiate activation of regulatory regions to activate gene expression that controls cell fate.

## Results

### HNF4A depletion in adult mouse liver alters histone modifications and chromatin accessibility

**Fig. 1:**
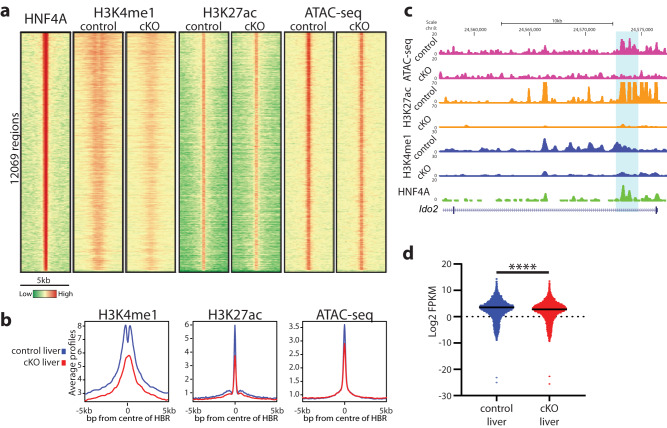
HNF4A loss leads to reduced histone modifications at HBRs in the mouse liver. **a** Heatmap displaying histone modifications (H3K4me1 and H3K27ac) and chromatin accessibility (ATAC-seq) at HNF4A bound regions (HBRs) in control and cKO livers. A 5 kb window is shown with the summit of HNF4A binding in the centre of each panel. Regions with high levels of each TF, histone modification or accessibility are red while low signal is represented by green. **b** Average profile plots are shown with the HBRs being at the centre. Control liver is in blue while *Hnf4a* cKO liver is in red. **c** Genome browser view of HNF4A target gene *Ido2* shows an example gene with reduced H3K4me1 (blue), H3K27ac (orange) and ATAC-seq (pink) in the cKO liver around the HNF4A bound region (green, highlighted). **d** RNA-seq data for genes associated with an HNF4A bound region show reduced overall expression in cKO livers (red) compared to control livers (blue). Mean is represented by black line. Significance was tested using a *t* test, ****p-value < 0.0001. *n* = 3 biologically independent samples.

We previously mapped HNF4A bound regions (HBRs) genome-wide in adult mouse liver and showed that the majority of HNF4A bound enhancers had a high level of H3K4me1^48,49^. Although a “hallmark” of a functional distal regulatory element is the presence of H3K4me1^32^, the mechanism that establishes and maintains H3K4me1 in relation to TF binding is not well understood. To address the connection between HNF4A binding and H3K4me1 maintenance, we used an *Albumin* promoter-driven Cre mouse strain (*Alb-CreER*^*T2*^) crossed with a floxed HNF4A allele to generate a tamoxifen-inducible, hepatocyte-specific deletion of HNF4A in adult livers (*Hnf4a* cKO liver)^10^ (Supplementary Fig. 1a). With tamoxifen treatment, HNF4A is effectively depleted in hepatocytes within 1 week as confirmed by western blot (Supplementary Figs. 1b and 7a) and we could examine the effects of acute HNF4A loss on histone modifications and chromatin in the adult liver. We performed genome-wide sequencing to map histone modifications (ChIP-seq and CUT&Tag) and chromatin accessibility (ATAC-seq) in control and *Hnf4a* cKO adult mouse livers (Fig. 1a). In agreement with recent papers^50,53^, we observed reduced levels of H3K4me1 in cKO livers at locations normally occupied by HNF4A (Fig. 1b). Of note, the Qu et al. dataset used a mouse model where HNF4A was deleted from liver cells during embryo development and it remained deleted as the mouse matured to adulthood, while our study maps chromatin changes that occurred following the loss of HNF4A in the adult, suggesting that HNF4A is required to maintain H3K4me1 at HBRs. While our previous data^48^ showed that loss of HNF4A caused a reduction in H3K27ac at a few sites that we examined, CUT&Tag on control and cKO livers identified changes on a genome-wide scale and showed reduction of H3K27ac at HBRs (Fig. 1b). Similarly, chromatin accessibility was modestly reduced at HBRs after deletion of *Hnf4a* (Fig. 1a, b). The genomic regions containing *Ido2* (Fig. 1c) and *Numa1* (Supplementary Fig. 1c) show examples of HNF4A-bound regions with reduced H3K4me1, H3K27ac and chromatin accessibility in the *Hnf4a* cKO livers. Of note, the composite profile plot of H3K4me1 ChIP-seq data shows higher H3K4me1 flanking HBRs in control livers but peaks centered at what would be the HBRs in cKO livers (Fig. 1b), indicating a shift in the positioning of the H3K4me1 marked nucleosomes that normally flank an HBR. This suggests that without HNF4A, the nucleosome positioning is disrupted, which may be a result of the fact that the loss of H3K4me1 reduces H3 eviction from the region^34^. This effect may relate to the loss of chromatin interactions at HNF4A bound enhancers that is observed with the loss of HNF4A in brush border epithelial cells^54^. Of note, due to methodological differences between ChIP-seq and CUT&Tag, nucleosome positioning information is not evident in the H3K27ac data. RNA-seq (see Supplementary Data 1 for full dataset) on control and *Hnf4a* cKO livers established that genes with an associated HBR were expressed at significantly lower levels in *Hnf4a* cKO livers (Fig. 1d) compared to control, supporting that depletion of HNF4A, and consequently reduced H3K4me1 and H3K27ac, diminish gene expression.

To ensure the robustness of our observations, we compared the adult mouse liver HNF4A ChIP-seq data from Reizel et al.^52^ to our HNF4A ChIP-seq data^48,49^ and observed that 95% of our ~12,000 HBRs matched regions identified as bound by HNF4A in their data (Supplementary Fig. 1d). While the Reizel dataset identified many additional HNF4A bound regions (over 36,000 total regions), we found H3K4me1 and H3K27ac were also reduced in *Hnf4a* cKO liver at this extended set of HBRs (Supplementary Fig. 1e).

In summary, the loss of HNF4A initiates a reduction of H3K4me1 and H3K27ac at HBRs, as well as a decrease in chromatin accessibility. HBR-associated gene expression is also reduced in mouse livers.

### HNF4A can maintain H3K4me1 in the absence of FOXA factors

**Fig. 2:**
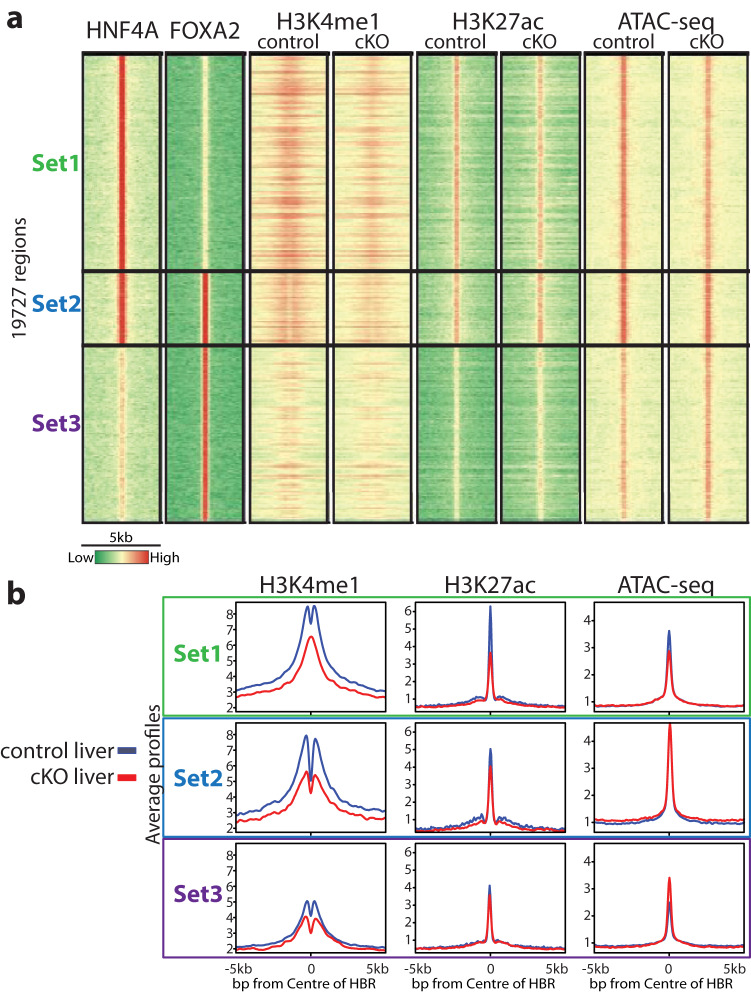
HNF4A maintains H3K4me1 at sites with HNF4A binding only and co-bound sites. **a** Heatmap displaying HNF4A bound and FOXA2 bound regions. Set1 represents all significant peaks that are only present in the HNF4A dataset, Set2 represents significant peaks shared in both datasets and Set3 represents significant peaks only called in FOXA2 dataset. The 5 kb region is centred around the TF bound region. **b** Average profile plots for H3K4me1, H3K27ac and ATAC-seq in control (blue) and cKO (red) livers for each set.

HNF4A is known to bind target regions with FOXA transcription factors^49,55^, a family of pioneer factors that contribute to chromatin accessibility and architecture. To investigate whether deletion of HNF4A affects histone modifications at FOXA2 sites, we looked at histone modification and ATAC-seq data centred around HNF4A and FOXA2 ChIP-seq data in adult liver^49^. We used our previously published^49^ high-confidence peaks for regions with only HNF4A bound (Set1), only FOXA2 bound (Set3) or both TFs bound (Set2) (Fig. 2a). All regions with HNF4A bound (Set1 and Set2) were identified as active regulatory elements with high H3K4me1, H3K27ac, and accessibility (Fig. 2b). In contrast, little H3K4me1 and H3K27ac were observed at Set3, where only FOXA2 is bound. As FOXA2 is a known pioneer factor^51^, it can be bound to closed or unmarked regions, suggesting additional factors may be required to activate these regions when needed. Interestingly, the regions with only HNF4A bound (Set1) showed reduction in both histone modifications and chromatin accessibility in the *Hnf4a* cKO liver (Fig. 2a, b) as well as a shift in H3K4me1 profile from nucleosomes flanking the HBR to nucleosomes present at the HBR (Fig. 2b). Set2, with both FOXA2 and HNF4A bound, showed a reduction of H3K4me1 but maintained nucleosome positioning with no drastic change in H3K27ac and accessibility in the *Hnf4a* cKO. Set3 had such low enrichment of the assayed histone modifications that the change between control and cKO is minimal. Expression of genes associated with HBRs was decreased more in Set1 and Set2 than in Set3 (Supplementary Fig. 2a).

Reizel et al.^52^ reported that livers lacking FOXA1, FOXA2 and FOXA3 (FOXA triple knock out -FTKO) had rapid necrosis and lethality due to loss of expression of critical liver genes caused by reduction in enhancer activity and binding of HNF4A at some FOXA bound regions. Interestingly, they identified a small subset of co-bound regions (~800) at which FOXA binding was required for HNF4A binding, and at these regions there was a decrease in both H3K4me1 and H3K27ac without FOXAs; however, many co-bound regions maintained HNF4A binding despite loss of FOXAs and these regions showed no change in assayed histone modifications^52^. We aligned the data for H3K4me1 and H3K27ac in FTKO livers, with our sets of HNF4A bound only, FOXA2 bound only or co-bound regions. In contrast to the *Hnf4a* cKO, we observed no global effect on histone modifications between control and FTKO livers at HBRs (Supplementary Fig. 2b–d). Thus, at the majority of binding sites, HNF4A remains bound, regardless of the presence of any FOXAs, suggesting that HNF4A, rather than FOXA proteins, is essential to maintain histone signatures.

### HNF4A expression in fibroblasts promotes a transition to epithelial cells

**Fig. 3:**
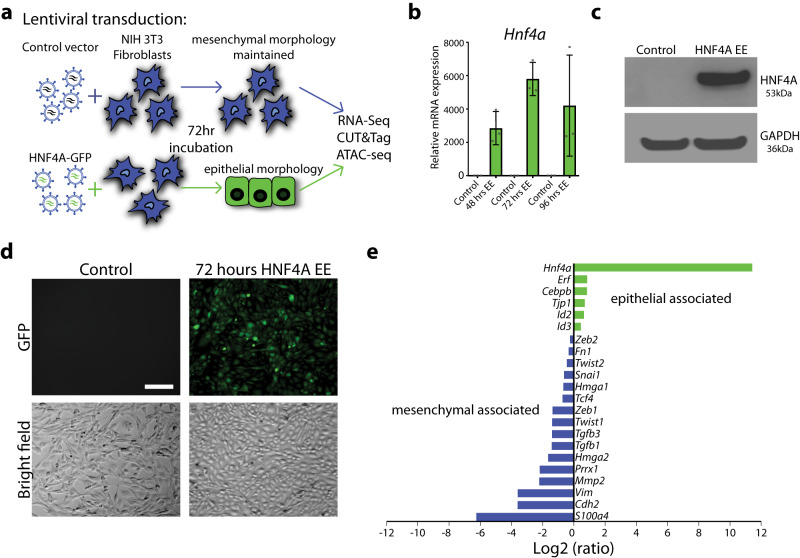
HNF4A ectopic expression causes morphological changes in mouse fibroblast cells. **a** Schematic overview of the experimental setup to produce 3T3 cells with ectopic expression (EE) of HNF4A. **b** RT-qPCR confirming increased *Hnf4a* expression 48, 72 and 96 h after transduction. Error bars, standard deviation. *n* = 3 technical replicates. **c** Western blotting confirms protein presence of HNF4A at 72 h post transduction. **d** GFP verifies HNF4A expression and bright field imaging demonstrates the morphological shift from mesenchymal to epithelial cells. Scale bar, 50 µm. **e** Bar plot with RNA-seq Log2 ratio of FPKM values associated with epithelial (green) and mesenchymal genes (blue) from control and EE cells.

Ectopic expression of master regulators can result in cellular reprogramming to drive cell type-specific gene expression patterns resulting in a phenotypic shift^56^. In this context, HNF4A, in combination with other factors^12,13,57^, is able to generate induced hepatocytes (iHep Cells) but the underlying mechanism that causes epigenetic and transcriptomic changes by HNF4A remains to be explored. To investigate the early events driven exclusively by HNF4A, we transduced mouse embryonic fibroblast (NIH 3T3 or 3T3) cells, which do not express HNF4A, with a construct carrying the mouse *Hnf4a* gene or an empty vector (Fig. 3a). Three days after transduction, ectopic expression of HNF4A (HNF4A EE) was confirmed by RT-qPCR (Fig. 3b and Supplementary Data 3) and western blot (Fig. 3c and Supplementary Fig. 6a), and we visually confirmed GFP expression in the cells using fluorescent microscopy (Fig. 3d). Importantly, the cells changed from their original elongated spindle shape, typical of mesenchymal cells, to the cobblestone-like shape, characteristic of epithelial cells (Fig. 3d). This aligns with previous studies in which HNF4A was termed a dominant regulator of the epithelial phenotype^58^. We collected RNA from cells transduced with each of the two vectors and used RNA-seq to investigate the transcriptional changes caused by HNF4A. RNA-seq analysis (log2 ratio > 0.5 or <−0.5 and *p*-value < 0.05) identified 5349 genes that are differentially expressed between the two conditions; 2668 genes were upregulated and 2681 genes were downregulated with HNF4A ectopic expression (Supplementary Fig. 3, see Supplementary Data 2 for full dataset). While HNF4A primarily functions as an activator of gene expression, the observation of almost equal numbers of genes induced and reduced suggests that major chromatin changes are occurring that accompany a cell fate change. This likely involves the induction of other transcriptional regulators. Since we observed morphological changes after expressing HNF4A (Fig. 3d), we examined the expression of key TFs and other genes associated with epithelial or mesenchymal cells. Epithelial related genes, such as *Id2*, *Id3*, *Erf*, *Tjp1*, and *Cebpb*, were upregulated while mesenchyme related genes, such as *Twist1, Snai1, Zeb1 and Vim*, were downregulated (Fig. 3e). This alongside the physical observations, indicate that these cells are undergoing a mesenchyme to epithelial shift. Of note, *FOXA1*, *FOXA2* and *FOXA3* were not induced in this system, making this model suitable to examine the exclusive role HNF4A has on shaping the epigenetic landscape.

### Ectopic expression of HNF4A can establish active enhancer modifications

**Fig. 4:**
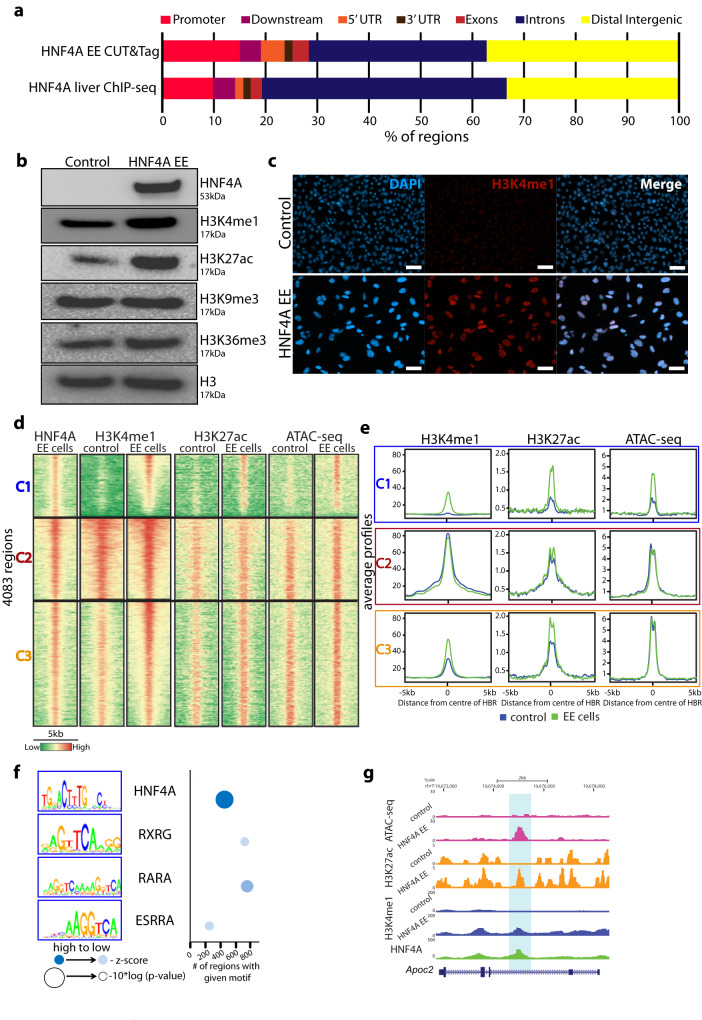
HNF4A regulates H3K4me1 levels in mouse fibroblast cells. **a** Bar graph showing breakdown of where HNF4A binds genomically in 3T3 cells expressing HNF4A (CUT&Tag) compared to binding in adult mouse liver (ChIP-seq). **b** Western blot showing HNF4A, H3K4me1, H3K27ac, H3K9me3, H3K36me3 in control and HNF4A EE 3T3 cells with total H3 as a loading control. **c** Fluorescent images confirming increased level of H3K4me1 (red) in HNF4A EE cells compared to control. DAPI staining (blue) captures each nucleus. Scale bar represents 50 µm. **d** Heatmaps showing all significant HNF4A bound regions from CUT&Tag and patterning of H3K4me1, H3K27ac and accessibility (ATAC-seq) in control and EE cells. Clusters (C1, C2 and C3) showing patterns fall into 3 categories. C1 represents the 942 regions that were newly activated with ectopic HNF4A expression. **e** Average profile plots for H3K4me1, H3K27ac and ATAC-seq for each cluster (C1-C3) for control (blue) and EE cells (green). **f** All non-redundant motifs identified in the newly activated C1 regions and plotted according to z-score, *p*-value and number of regions with the given motif. **g** *Apoc2* gene shows changes in H3K4me1, H3K27ac and accessibility (ATAC-seq) with HNF4A expression in 3T3 cells. HNF4A bound region is highlighted.

We used CUT&Tag to identify where HNF4A bound in the induced system and identified 4083 HNF4A-bound regions in these cells preferentially at intronic and distal intergenic regions, similar to the adult liver (Fig. 4a). GO analysis of genes associated with all HBRs showed enrichment for metabolic associated functions as expected (Supplementary Fig. 4a). Comparison of the HBRs in the expression system to the adult liver found only 839 regions (20.5% of regions bound in HNF4A expressing 3T3s) represented in both datasets (Supplementary Fig. 4b) although HNF4A binding motifs were similar (Supplementary Fig. 4c). This highlights that many HBRs are unique across cell types and HNF4A does not bind all genomic regions where a motif is evident. These common 839 regions are associated with genes enriched for metabolic processes (Supplementary Fig. 4d). RNA-seq analysis of genes associated within this subset of bound regions show significantly increased expression in HNF4A-expressing 3T3 cells and significantly decreased expression in *Hnf4a* cKO livers compared to their respective controls (Supplementary Fig. 4e).

We used the 3T3 cells ectopically expressing HNF4A to examine the deposition of H3K4me1 and other epigenetic changes. After 3 days of HNF4A ectopic expression, the overall level of H3K4me1 was increased (~23%) as observed by both western blotting (Fig. 4b and Supplementary Fig. 6b) and immunofluorescence (Fig. 4c). We examined levels of other histone modifications by western blotting and found H3K27ac increased (~59%) alongside H3K4me1 in cells expressing HNF4A (Fig. 4b). Other histone modifications did not change significantly. We mapped genome wide epigenetic changes caused by HNF4A expression using CUT&Tag for H3K4me1 and H3K27ac, and ATAC-seq to examine accessibility, in both conditions. We clustered the HBRs based on H3K4me1 enrichment and identified three clusters (C1, C2 and C3) with C1 regions showing low enrichment of active histone modifications and low chromatin accessibility prior to HNF4A expression (Fig. 4d, e). These 942 regions became newly activated with high H3K4me1, H3K27ac and ATAC-seq signal only after HNF4A expression and motif analysis showed HNF4A as the most significantly enriched (Fig. 4f).

Similar to C1, C3 (1905 regions) showed a substantial increase of H3K4me1 and H3K27ac around HBRs, although low levels of histone modification were present in control samples suggesting that these regions were already accessible. An example is shown in the *Apoc2* gene, which was significantly downregulated in the cKO liver and upregulated in the cells expressing HNF4A (Fig. 4g and Supplementary Data 1, 2). C2 (1236 regions) maintained the same levels of histone modifications and accessibility following HNF4A expression. We also looked at all H3K4me1 regions, regardless of HNF4A presence in 3T3 cells, and found 3207 regions (CL2) that gained the mark in cells expressing HNF4A (Supplementary Fig. 4 f, g). It is noteworthy that while only a proportion of these regions are bound by HNF4A three days after transduction, motif analysis found JUND and HNF4A as the most significant in this cluster with the HNF4A motif represented in over 96% of these newly methylated regions (Supplementary Fig. 4h). This suggests that binding by HNF4A may be transient at some locations.

Our data suggests that binding of HNF4A in cells that do not normally express HNF4A causes an increase in H3K4me1, H3K27ac and chromatin accessibility and can establish H3K4me1 to activate a unique set of regulatory regions that initiate a shift from a mesenchymal to epithelial cell state.

### HNF4A interacts with the MLL4 complex to establish H3K4me1

**Fig. 5:**
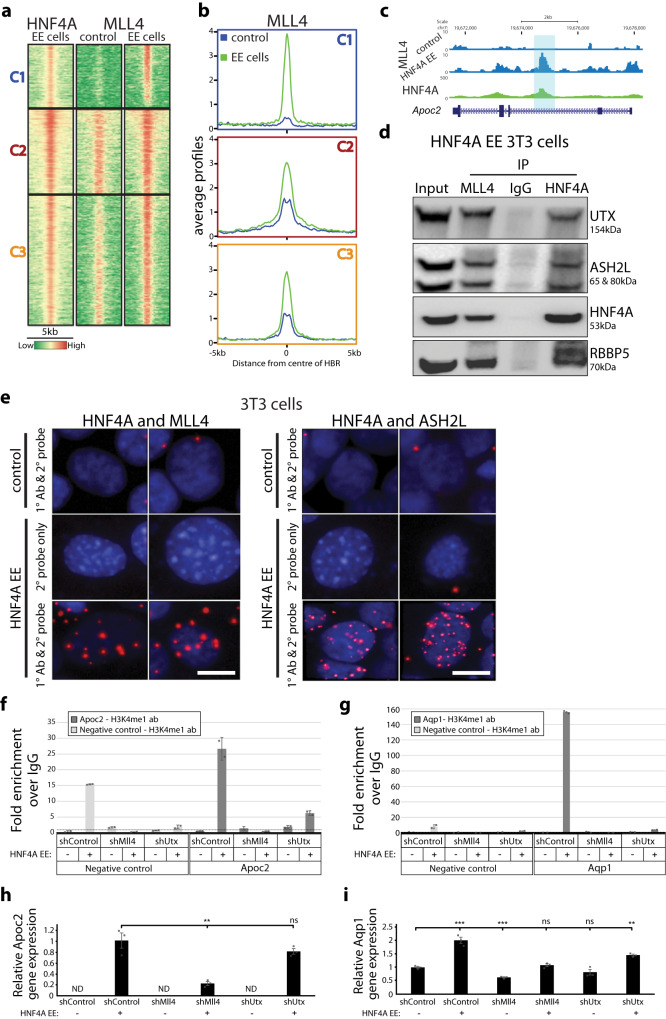
HNF4A recruits the MLL4 complex to establish H3K4me1. **a** Heatmap showing the same clustering as in Fig. 4d with MLL4 CUT&Tag in control and EE cells in a 5 kb window around HNF4A bound regions. **b** Average profile plots for MLL4 in control (blue) and EE cells (green) across the 3 clusters. **c** Genome browser depiction of *Apoc2* shows MLL4 binding at site of HNF4A binding. **d** Co-immunoprecipitation using antibodies for HNF4A, MLL4 and IgG (as negative control) in cells ectopically expressing HNF4A followed by western blot analysis with MLL4 complex proteins (UTX, ASH2L and RBBP5) or HNF4A shows interaction is occurring between HNF4A and MLL4 proteins in 3T3 cells. **e** Proximity ligation assay (PLA) in control and HNF4A EE 3T3 cells. Top two panels show negative controls while the bottom panel shows positive signals (red) identify interactions occurring between HNF4A and MLL4 (left) or HNF4A and ASH2L (right). DAPI stained nuclei are shown in blue. Scale bars, 10 µm; Ab, antibody. **f**–**i** 3T3 cells were transduced with control, *Mll4*, or *Utx* shRNA and after 24 h, the cells were transduced with HNF4A or empty construct. The cells were further incubated at 37 °C for 48 h before performing the ChIP-qPCR and RT-qPCR analyses. The ChIP-qPCR for H3K4me1 in control shRNA-transduced 3T3 cells show increased H3K4me1 at HBR regions linked to *Apoc2* (**f**) and *Aqp1* (**g**) when HNF4A was overexpressed. However, H3K4me1 enrichment levels were significantly reduced when either *Mll4* or *Utx* was knocked down. A dotted line represents a fold enrichment of 1. Using RT-qPCR for *Apoc2* (**h**) and *Aqp1* (**i**), relative gene expression levels were measured. The bars display standard deviation (**f**, **g**) or standard error of mean (**h**, **i**). The statistically significant *p*-values were calculated by a *t* test. ***p < 0.001; **p < 0.05; ND, not detected; ns, not significant. *n* = 2 (**f**, **g**) or 3 (**h**, **i**) technical replicates.

MLL3 and MLL4 catalyze H3K4 monomethylation and are recruited to target enhancers by TFs and pioneer factors^37,38,59^. To understand the role of MLL4 in HNF4A’s establishment of H3K4me1, we completed CUT&Tag for MLL4 on cells ectopically expressing HNF4A. Using the clusters identified in Fig. 4, all regions showed increased MLL4 binding following HNF4A expression (Fig. 5a, b). The *Apoc2* gene locus shows the newly bound MLL4 at the HBR (Fig. 5c). This suggests that HNF4A binding to these regions promotes recruitment of MLL4. The C1 cluster shows a dramatic increase in MLL4 binding. Interestingly, while the C2 cluster showed very strong H3K4me1 signal, regardless of HNF4A presence, the MLL4 signal increased further with HNF4A binding. The C3 cluster, with low H3K4me1 and MLL4 bound in control 3T3 cells, had both increased with HNF4A binding (Figs. 4d, 5b). This suggests that in these two clusters the regions are already active but binding of HNF4A augments their activity.

MLL4 is known to exist at enhancers in a complex with UTX (KDM6A), ASH2L, and RBBP5^38^. To further support our findings, we evaluated publicly available ChIP-seq data in HepG2 cells (ENCODE), a human hepatoma cell line. The HepG2 data for HNF4A, histone modifications, and MLL4 complex proteins suggested that HNF4A binds at the same regions as the complex proteins (ASH2L and UTX) and histone acetyltransferase (p300), which respectively establish H3K4me1 and H3K27ac on nearby nucleosomes (Supplementary Fig. 5a).

Based on the genomic data showing MLL4 bound alongside HNF4A, we hypothesized that HNF4A interacts with the MLL4 complex and recruits it to enhancers to establish H3K4 methylation. An interaction between HNF4A and the MLL4 complex proteins, ASH2L, RBBP5 and UTX, was confirmed by co-immunoprecipitation in 3T3 cells ectopically expressing HNF4A (Fig. 5d and Supplementary Fig. 6c). These interactions were confirmed to also occur in HEK293T cells (overexpressing HNF4A) as well as in mouse livers where HNF4A is endogenously expressed (Supplementary Figs. 5b, 6c). Proximity ligation assay (PLA) validated the association between HNF4A and MLL4 or HNF4A and ASH2L proteins in situ in 3T3 cells (Fig. 5e and Supplementary Fig. 5c) and HEK293T cells (Supplementary Fig. 5d, e) ectopically expressing HNF4A.

To explore the functional dependence of the HNF4A-MLL4 complex, we used shRNA knockdown to reduce expression of a component of MLL4 complex (either MLL4 or UTX) in the 3T3 cells, followed by ectopic expression of HNF4A. We then examined H3K4me1 levels and gene expression of the HNF4A targets, *Apoc2* and *Aqp1*. Ectopic expression of HNF4A in control shRNA-transduced cells increased H3K4me1 at the HBRs. Moreover, their gene expression levels were increased. In contrast, treatment with MLL4 shRNA resulted in a significant reduction of the H3K4me1 at the HBRs and gene expression of *Apoc2* and *Aqp1* (Fig. 5f–i and Supplementary Figs. 5f–i and 7b–d) (see also Supplementary Data 3 for details). We observed a similar effect in UTX shRNA treated cells. Overall, our data support that HNF4A-mediated induction of H3K4me1 at HBRs and the subsequent gene transcription depend on the MLL4 complex.

Together, our findings suggest that the physical interactions between HNF4A and proteins in the MLL4 complex, in vitro and in vivo, act to recruit MLL4 to enhancers to establish H3K4me1 causing a cascade effect that activates the enhancer to induce gene expression. Our data indicate that HNF4A is required for the recruitment of this complex to enhancers to establish and maintain a transcriptionally permissive state.

## Discussion

Cell fate is determined by direct interactions between master regulators and cell-type-specific enhancers^51^. While TFs like HNF4A regulate gene expression by influencing the epigenetic landscape^48,50^, the molecular mechanism is unknown. Here, we propose a mechanism whereby HNF4A, a regulator of hepatocyte identity, interacts with the epigenetic modifier MLL4 to deposit H3K4me1 at HBRs.

While a concurrence of HNF4A binding and H3K4me1 has been well documented^48–50,52,60^, we show that HNF4A is essential for maintaining H3K4me1 at HBRs, and consequently H3K27ac, in the adult mouse liver. Within one week after the loss of HNF4A, H3K4me1 and H3K27ac levels are substantially reduced at HBRs. Moreover, ATAC-seq levels are reduced, indicating that changes in chromatin structure are initiated. We further show, using an in vitro system, that ectopic expression of HNF4A can augment H3K4me1 and H3K27ac and, at a subset of sites, can dramatically increase chromatin accessibility alongside the active histone modifications. A recent publication shows that HNF4A is required for establishing and maintaining the chromatin looping that ensures the enhancers are engaged with a promoter to activate transcription^54^. Together these data indicate that HNF4A establishes enhancers as active, with open chromatin marked by H3K4me1 and H3K27ac, and facilitates how these enhancers interact with the nearby promoter.

The active histone modification H3K27ac at gene regulatory regions is dependent on the presence of H3K4me1, indicating H3K4me1 defines the future active enhancer^30,34^. MLL4 serves as a main H3K4 mono-methyltransferase for enhancer activation^35,36,38^ and exhibits cell type and differentiation stage specific binding^37,41^. It has been shown that MLL4 is required for H3K27ac, Pol II, and mediator recruitment to enhancers^37^ and recent studies showed that the MLL4 complex recruits and activates p300 activity, involving UTX, at enhancers, resulting not only in methylation at H3K4 but also acetylation on H3K27 at target chromatin^41,61^. Our studies demonstrate that HNF4A associates with the MLL4 complex components, ASH2L, UTX and RBBP5. MLL4 has also been shown to co-localize with lineage-specific TFs such as C/EBPβ, PPARγ, EBF1 and GR at enhancers to ensure increased expression of cell-type-specific genes that establish lineage maturity^37^. Ectopic expression of C/EBPβ in preadipocytes recruited MLL4 to establish adipogenic enhancers. Similarly, we found that HNF4A-bound regions correlated with MLL4 binding in cells where HNF4A was ectopically expressed. In addition, using two different methods we confirmed that HNF4A and the MLL4 complex physically interact in mouse liver and two cell lines expressing HNF4A. Moreover, HNF4A has been shown to interact with the histone acetyltransferase, p300^62,63^, although whether this is direct or through the MLL4 complex has not been confirmed. Thus, HNF4A joins a growing class of TFs that act by recruiting the required machinery to initiate and maintain enhancer histone modifications.

**Fig. 6:**
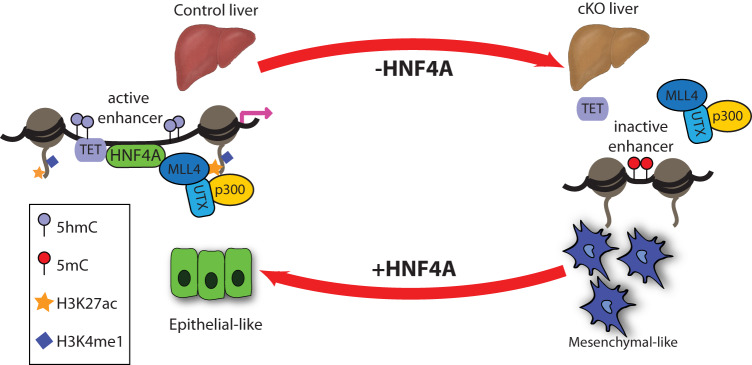
Interplay between HNF4A and epigenetic machinery required for gene expression in vitro and in vivo. HNF4A is required for the establishment and maintenance of the active enhancer state in vivo (top) and in vitro (bottom). HNF4A binding sites at enhancers in control livers show high H3K4me1 (dark blue) and H3K27ac (yellow) with chromatin accessibility (dark grey). HNF4A (green) interacts and recruits the MLL4 complex to enhancers and decorates enhancers with these active modifications resulting in higher gene expression (pink arrow). In cKO livers, HNF4A depletion results in loss of HNF4A mediated recruitment of MLL4 complex to enhancers (shown on right) and loss of active histone modifications resulting in reduction in gene expression. In 3T3 cells (bottom), ectopic expression of HNF4A in 3T3 cells results in morphological changes from mesenchymal to epithelial state resulting in histone modifications chromatin accessibility exhibited by cells in normal liver.

Previously, we determined that HNF4A interacts with TET proteins to maintain hydroxymethylation, an active DNA modification at enhancers^48^. Given that HNF4A interacts with the MLL4 complex, TET proteins and histone acetyltransferases, HNF4A appears to function to coordinate the active chromatin state. Based on these findings, we propose a stepwise model (Fig. 6) where HNF4A binds genomic regions containing the HNF4A motif, recruits TET proteins and MLL4, resulting in hydroxymethylation of cytosine and monomethylation of H3K4. With p300 being recruited by the MLL4 complex, the nucleosomes are then marked by H3K27ac. These modifications cause changes in charge to the histones, which contributes to loosening of the DNA bound to the nucleosome, increasing accessibility and allowing further binding by other factors. Overall, these findings suggest that HNF4A facilitates the formation of a transcriptionally permissive hub by recruiting the epigenetic machinery to drive cell-type-specific gene expression.

HNF4A expression has previously been proposed to impact chromatin structure: in fibroblast cells, ectopic expression of HNF4A-induced chromatin accessibility to regulate the expression of corticoid binding globulin (CBG) and α1-antitrypsin (α1AT)^64^. In addition, ectopic expression of HNF4A in normal oesophageal cells showed that HNF4A drives the formation of open chromatin within 48 h, although HNF4A binding was not confirmed^65^. While our findings focused on H3K4me1 induction, there was a reduction in accessibility in the *Hnf4a* cKO livers around HBRs, and while most regions bound by HNF4A in cells ectopically expressing HNF4A were already open, a portion gained accessibility at bound sites. This indicates that HNF4A can promote the open chromatin state.

Due to this ability to affect chromatin structure, it was suggested that HNF4A acts as a pioneer factor^50,65^. Pioneer factors are defined by their ability to identify their target sequences in closed chromatin and facilitate remodelling to increase accessibility^66,67^. Pioneer factors can scan DNA sequences on the surface of a nucleosome to access silent chromatin and initiate opening that allows other TFs, proteins and histone modifiers to alter the chromatin landscape. While we have shown that HNF4A expression changes accessibility at a subset of regions and that it is capable of recruiting the MLL4 complex and encouraging histone modifications that activate an enhancer, HNF4A is not bound to closed chromatin. Well established pioneer factors, such as FOXA1, FOXA2, PAX7, GATA3 or GATA6, bind regions in unmarked chromatin^65,68–70^. It has been proposed that this binding represents the scanning function of the pioneer factors^67^. Since the vast majority of HBRs have associated H3K4me1 and H3K27ac, HNF4A likely does not have this scanning function in closed chromatin. While HNF4A can efficiently affect H3K4me1 and H3K27ac levels, it has been shown that HNF4A is unable to bind nucleosomes^71^, a defining feature of pioneer factors. While there is a possibility that HNF4A binding causes immediate opening of chromatin and therefore is only seen at open chromatin, the lack of other defining features of a pioneer factor suggest that additional factors are required to facilitate opening of chromatin. Further investigation with well-timed experiments will be needed to define the players required.

HNF4A is known to cooperate with the FOXA family and GATA6 of pioneer factors^12,49,55^. Using mouse livers depleted for FOXAs, a subset of regions was identified where HNF4A and FOXA normally co-bound but HNF4A binding was also lost with loss of FOXAs^52^. At these sites alone, there was a reduction in H3K4me1 and H3K27ac in TFKOs while the majority of normally co-bound sites maintained strong HNF4A binding and the histone modifications remained intact regardless of loss of FOXAs. This indicates that HNF4A can maintain H3K4me1 and H3K27ac in livers in the absence of FOXA. Supporting this, we also observed that HNF4A ectopic expression induced chromatin accessibility in cells lacking FOXA expression. However, other pioneer factors may facilitate chromatin opening since within the regions newly activated with the addition of HNF4A, we found motifs for other TFs such as RXRG, RARA and ESRRA indicating that additional mechanisms are likely involved.

It is difficult to correlate the expression of single genes to specific enhancers as precise mapping of enhancers to genes is often inaccurate. Also, most genes are regulated by multiple enhancers since redundancy is built into gene regulatory mechanisms. In our studies, we observed similar effects of HNF4A on overall genomic changes. However, it is noteworthy that the two systems we used show a fundamental difference. In the adult liver HNF4A was removed from an already differentiated state while in the 3T3 cells expression of HNF4A caused a cell fate transition in a fibroblast cell line. These differences account for distinct HNF4A-bound regions and how gene expression is managed in the two systems. For example, *Apoc2* is downregulated in the *Hnf4a* cKO mouse liver where HNF4A is bound at the *Apoc2* promoter region. However, there are no changes in the chromatin accessibility or histone modifications, likely reflecting that other factors are present at promoters. In contrast, *Apoc2* is upregulated in 3T3 cells ectopically expressing HNF4A, where HNF4A is bound at an intronic enhancer and levels of histone modifications are increased. It is interesting that both states result in an expression change for the gene. In contrast, *Ido2* expression was drastically reduced in the *Hnf4a* cKO livers while it was not altered (remained off) in the cell system. These examples highlight how distinct gene regulatory elements can produce similar changes in gene expression due to tissue-specific facets. Thus, changes in overall genomic patterns may not be generalized to all genes.

In summary, our work demonstrates the essential role that HNF4A plays in liver by establishing and maintaining the epigenetic landscape via direct interaction with the MLL4 complex. Our work also highlights the role of HNF4A in regulating MET and the epithelial phenotype of cells, and we provide a mechanism for HNF4A-controlled gene regulation. These findings provide a greater understanding of how the aberrant epigenetic program, upon loss or reduced expression of HNF4A, can contribute to disease progression, such as in hepatocellular carcinoma.

## Methods

### Mice and cell preparation

All mouse protocols were approved for ethics by the Animal Care Committee, University of British Columbia. We have complied with all relevant ethical regulations for animal use. For HNF4A and FOXA2 ChIP-seq, livers from 8-week-old female mice (C57BL6/J) were collected. For *Hnf4a* cKO mice, mice with a tamoxifen inducible, *Albumin* promoter-driven Cre (*Alb-CreER*^*T2*^) were crossed with *Hnf4a*^fl/fl^ mice^10^ and tamoxifen (2 mg/ml, intraperitoneal, subcutaneously) was administered to adults every other day until day 7 when the mice were euthanized and livers were collected. Control mice for the cKO were *Hnf4a*^fl/fl^, Cre negative with no tamoxifen. The HNF4a knockout animal studies and procedures were carried out in accordance with the National Cancer Institute Animal Care and Use Committee. Mice were maintained in a pathogen free animal facility with a 12-h light/dark cycle.

### Cell culture and lentiviral transduction

Mouse fibroblast (NIH 3T3 or 3T3), human embryonic kidney (HEK293T), and human hepatoma (HepG2) cells were purchased from the American Type Culture Collection (Manassas, VA). Cells were cultured in DMEM (with high glucose and pyruvate) media (Gibco^TM^) supplemented with 10% fetal bovine serum (FBS) (Gibco^TM^) and 1% penicillin and streptomycin (Gibco^TM^). All cells were maintained in humidified incubators with 5% CO_2_ at 37 °C. A lentivirus construct with mouse *Hnf4a-*IRES-eGFP was used to ectopically express Hnf4a (isoform 2, 474 a.a.). The packaging/envelope vectors pCMV-dR8.74, pCMV-VSV-G, and pRSV-Rev were kindly gifted by Dr. Andrew Weng (Terry Fox Laboratory). For lentiviral packaging, HEK293T cells were cultured on 10 cm plates using DMEM supplemented with 10% FBS. The co-transfection of lentiviral and packaging plasmids was achieved using polyethyleneimine (PEI). The media was replaced after 24 h and the supernatant was collected 48 h post-transfection. Cells were grown in 6 well plates and lentiviral supernatant was titrated to transduce >95% of the cells. The media was replaced after 24 h and cells were collected 72 h (3 days) post-transduction. HNF4A expression was confirmed by detecting GFP expression, RT-qPCR and western blot analysis.

### In situ proximity ligation assay (PLA)

NIH 3T3 and 293T cells were cultured on coverslips and washed twice with PBS before fixation and permeabilization. Cells were fixed with 4% paraformaldehyde (Sigma-Aldrich) at room temperature for 15 minutes and then permeabilized with Triton-X (0.1% v/v in PBS). PLA was performed according to the manufacturer’s instructions, using Duolink in situ PLA kit (Sigma-Aldrich catalogue #DUO 92102). Images were captured using a Zeiss AxioImager M2.

### Co-immunoprecipitation (Co-IP)

Cells were lysed using CHAPS buffer according to the manufacturer’s protocol (FIVEphoton Biochemicals). In brief, 300 µl ice cold CHAPS buffer with 1x protease inhibitor cocktail tablets (PIC, ThermoFisher) was added to each 10 cm cell culture dish. After scraping cells from the plate, cells were transferred to microcentrifuge tubes and incubated on ice for 10 min. Cell lysates were centrifuged for 15 min at 4 °C at max speed. The supernatant was used for further IP experiments. The lysates were incubated with 2.5 µg of HNF4A or MLL4 antibody at 4 °C overnight. The next day, 20 µl of protein A/G beads (ThermoFisher) was added to the lysates, and samples were rotated for 30 minutes at room temperature. Bead complexes were centrifuged at 4000 rpm for 5 min and washed 3 times using 300 µl CHAPS lysis buffer. SDS gel loading buffer was added to the beads and heated at 65 °C. The resulting proteins were separated and transferred according to the Western blot protocol below and analyzed using antibodies specified in Supplementary Table 1.

### Western blot

Cell lysis was performed using ice-cold lysis buffer (Cell Signalling Technology) supplemented with 1x PIC. Pierce BCA protein assay kit (ThermoFisher) was used for protein quantifications. An equal amount of protein (50 µg) was separated using NuPAGE 4-12% gels (ThermoFisher) and transferred to PVDF membranes. For the MLL4 blot, 3–8% Tris-acetate gels (ThermoFisher) were used alongside corresponding buffers. After blocking with 5% non-fat milk in TBST for 1 h, membranes were incubated with primary antibodies in 5% milk overnight at 4 °C. The membranes were washed with TBST 3 times for 10 min and incubated with HRP-conjugated secondary antibodies in 5% milk at room temperature for 1 h. HRP activity was detected using Pierce ECL Western Blotting Substrate (ThermoFisher). Antibodies used in this manuscript are listed in Supplementary Table 1.

### ChIP-seq

Chromatin immunoprecipitation (ChIP) was performed using pulverized frozen livers^72^. The liver samples were fixed with 1 ml 1% formaldehyde in PBS for 10 minutes at room temperature, quenched with glycine (final concentration 125 mM) for 5 minutes, pelleted by centrifugation (3000 rpm for 3 minutes at 4 °C) and washed twice with PBS for 5 minutes each. Following removal of PBS, cells were lysed for 15 minutes on ice using a volume of cold cell lysis buffer (with final concentrations of 10 mM of Tris‐HCl [pH 8], 10 mM NaCl, 3 mM MgCl_2_, and 0.5% Nonidet P‐40, supplemented with 1x PIC) equal to 5 times the cell pellet. Nuclei were pelleted by centrifugation (13200 rpm, 5 minutes, 4 °C) and then nuclear lysis buffer (50 mM of Tris‐HCl [pH 8], 5 mM of EDTA, 1% SDS, and 1× PIC) was added at a volume of 4 times the nuclear pellet and incubated on ice for 60 min. To shear the chromatin, sonication was performed in a cuphorn Q Sonica Sonicator Q700 for a total of 10 minutes (30-s cycles on/off) to generate 200-500 bp fragments. A volume of chromatin (equal to approximately 1 million cells or 50 µl or up to 10 µg depending on the sample) chromatin was diluted with a volume of ChIP dilution buffer (16.7 mM of Tris‐HCl [pH 8], 167 mM of NaCl, 0.01% SDS, 1.1% Triton X‐100, 1.2 mM of EDTA, and 1× PIC) equal to 4 volumes of chromatin, and 20 µl Pierce Protein A/G Ultralink beads (ThermoFisher) and PIC were added and rotated at 4 °C for 1 h to clear the chromatin. Beads and chromatin were spun at 4000 rpm for 2 min and the solution containing the chromatin was removed from the beads and added to fresh tubes with 3 µg of transcription factor antibody or 2 µg histone modification antibody (Supplementary Table 1) and rotated at 4 °C overnight. Protein A/G beads were added to the samples in the morning and rotated for 4 h at 4 °C. A/G bead complexes were washed with rotation in 0.5 ml low salt wash (20 mM of Tris‐HCl [pH 8], 0.15 M of NaCl, 2 mM of EDTA, 0.1% SDS, and 1% Triton X‐100), high salt wash (20 mM of Tris‐HCl [pH 8], 0.5 M of NaCl, 2 mM of EDTA, 0.1% SDS, and 1% Triton X‐100), lithium chloride wash (10 mM of Tris‐HCl [pH 8], 0.25 M of LiCl, 1 mM of EDTA, 1% Nonidet P‐40, and 1% sodium deoxycholate), and Tris‐EDTA buffer (twice) for 5 min each with centrifugation at 4000 rpm for 2 min to settle beads. Samples were eluted from beads using 125 µl of 0.1 M of sodium hydroxycholate in 1% SDS for 15 min at room temperature followed by centrifugation at 6000 rpm for 2 min. Elution was repeated with a second volume of 125 µl. To the 250 µl of eluted chromatin, 10 µl of 5 M NaCl, 10 µl 1 M Tris-HCl (pH 6.5), 5 µl 0.5 M EDTA, 2.5 µl 10 mg/ml RNAse A (Invitrogen) and 2 µl 10 mg/ml proteinase K (Ambion) were added to the eluted samples followed by overnight incubation at 65 °C. DNA was extracted using a standard phenol-chloroform extraction (using phenol-chloroform twice followed by just chloroform) and the final aqueous phase precipitated 1:10 with 0.3 M sodium acetate, 1:100 glycogen (ThermoFisher, 20 µg/ml) and 2.5x volume of anhydrous ice cold ethanol. Following incubation at −80 °C for 1 h, samples were spun down at 13200 rpm for 30 minutes at 4 °C. Pellets were washed with 70% ethanol, spun down 15 min at max speed and dried at room temperature. The pellets were resuspended in nuclease-free water and used directly for qPCR analysis and library construction.

### Cleavage under targets and tagmentation (CUT&Tag)

CUT&Tag was performed using ~500,000 cells per assay^73^. For liver samples, 3-5 mg of liver tissues was pulverized using liquid nitrogen and used for CUT&Tag. 3T3 cells and pulverized liver samples were washed with 1.5 ml wash buffer (20 mM HEPES pH 7.5, 150 mM NaCl, 0.5 mM spermidine, and 1x PIC) and resuspended in 1 ml wash buffer. Next, 10 µl of prepared Concanavalin-A coated magnetic beads (Bangs Laboratories) were added to each sample and incubated for 15 min at room temperature. A magnet was used to pull down beads for 2 min, unbound solution was removed and bead-bound cells were resuspended in 100 µl wash buffer containing 0.05% digitonin (ThermoFisher), 2 mM EDTA and the histone modifications or HNF4A antibody (0.5 or 1 µg respectively, Supplementary Table 1). Samples were incubated overnight at 4 °C, washed using the magnet stand, and incubated with secondary antibody (Supplementary Table 1) diluted in Dig-Wash buffer (1:50) for 1 h at room temperature. Following 2 washes with Dig-Wash buffer for 5 min each, samples were incubated with 100 µl of 1:200 pA-Tn5 adapter complex diluted in Dig-300 buffer (0.01% Digitonin, 20 mM HEPES, pH 7.5, 300 mM NaCl, 0.5 mM Spermidine, PIC) for 1 h at room temperature, and then samples were incubated again with Dig-300 buffer containing 10 mM MgCl_2_ at 37 °C for 1 h. The tagmentation was stopped by adding 16.6 mM EDTA, 0.1% SDS, and 50 µg Proteinase K and incubated for 1 h at 50 °C. DNA was extracted by the phenol-chloroform method as described in the ChIP-seq section. Sequencing libraries were generated using barcoded primers^74^. The libraries were amplified for 14 cycles using a thermocycler. DNA purification was achieved using sparQ PureMag Beads (Quantabio). Libraries were sequenced at the University of British Columbia (now SBME Sequencing Facility).

### ATAC-Seq

For NIH 3T3 ATAC-seq^75^, 100,000 cells were used per experiment and for liver samples 3 mg of pulverized tissue was used as starting material. Following centrifugation at 500 g for 5 min at 4 °C, cells were washed with cold PBS and then resuspended in cold lysis buffer (10 mM Tris-HCl, pH 7.4, 10 mM NaCl, 3 mM MgCl2, 0.1% NP-40). Cells were lysed on ice for 15 min and then spun down at 500 g for 10 min at 4 °C. After removing the supernatant, the nuclei were resuspended in transposition reaction mix (25 µl TD 2x reaction buffer, 2.5 µl Nextera Tn5 Transposase, 22.5 µl nuclease free water). The reaction was incubated for 30 min at 37 °C. DNA was purified using a PCR purification kit (ThermoFisher) and eluted in elution buffer (10 mM Tris buffer, pH 8). DNA was amplified for 10 cycles using a thermocycler and barcoded primers^74^. DNA purification was achieved using sparQ PureMag Beads (Quantabio). Libraries were sequenced at the University of British Columbia (now SBME Sequencing Facility).

### CUT&Tag, ATAC- and ChIP-seq data processing and analysis

The sequencing reads were mapped to mm10 genome by the BWA aligner^76^ version 1.1.4. Duplicate reads were marked using Picard version 2.1.1 (https://github.com/broadinstitute/picard) on Illumina base space. The encode_task_filter.py script from the ENCODE ChIP-seq pipeline 2 (https://github.com/ENCODE-DCC/chip-seq-pipeline2/blob/master/src/encode_task_filter.py) was used to filter the Binary Alignment Map (BAM) files. The encode_task_filter.py script eliminates low quality (MAPQ < 5), unpaired and unmapped reads using SAMtools version 1.3^77^ with the SAM flag filter “-F 1804”. Peaks were called using MACS2 with parameters “-p 0.01 -f BAMPE -g mm” for CUT&Tag data, and “-p 0.01 -g mm --nomodel --shift −75 --extsize 150” for the ATAC-seq data^78^. Bigwig (bw) files used for downstream analysis were generated using MACS2 bdgcmp by using the fold enrichment option for the ATAC-seq data. All bed and bw files were filtered using the ENCODE Blacklist (https://sites.google.com/site/anshulkundaje/projects/blacklists). Bed and bigwig files were analyzed using Cistrome^79^, Galaxy^80^, integrative genome browser (IGV)^81^. The genomic maps for individual genes were generated using the UCSC genome browser https://genome.ucsc.edu/. Motif analysis was performed using the Seqpos tool^79^ by scanning 300 bp spanning the HNF4A, H3K4me1 and MLL4 peak summits. k means clustering and H3K4me1 and MLL4 differential binding analysis was performed using ChAsE^82^. ChIP-seq and CUT&Tag heatmap were generated using ChAsE^82^ and profile plots for the same data were generated using the sitepro tool in Cistrome. Genomic location analysis of HNF4A, H3K4me1 and MLL4 was performed using the CEAS tool^83^. Genes associated with regulatory regions were identified using GREAT^84^.

### RNA-seq and analysis

RNA samples from cell lines and pulverized livers were extracted using TRIzol (ThermoFisher) reagent using the manufacturer’s protocol. Three biological replicates were used for RNA extraction followed by sequencing at the University of British Columbia (now SBME Sequencing Facility). Sequencing reads were aligned to mm10 using the STAR aligner^85^. Cufflinks^86^ was used to calculate fragments per kilobases of exon per million reads (FPKM) values. For the downstream analysis of RNA-seq data, log2 ratio ≥ 0.5 or ≤−0.5 and *p* value < 0.05 were used. See Supplementary Data 1 and 2.

### Statistical and Reproducibility

RT-qPCR results were calculated as mean ± SEM or mean (SD) derived from three experiments and two replicates were used for ChIP-qPCR. The statistical significance (*p*-value) was calculated with a Student’s *t* test using GraphPad or Microsoft Excel software. Western blots, Co-IP and PLA experiments were completed multiple times to ensure reproducibility with a representative shown. ChIP-seq, CUT&Tag and ATAC-seq were all completed only once although qPCR was used for validation (not shown). RNA-seq was done on 3 biological replicates.

### Reporting summary

Further information on research design is available in the Nature Portfolio Reporting Summary linked to this article.

### Supplementary information

Supplementary Information

Description of additional supplementary files

Supplementary Data 1

Supplementary Data 2

Supplementary Data 3

Reporting Summary

## Data Availability

The data generated in this manuscript is available through GEO accession numbers: GSE210842 (RNA-seq) and GSE211123 (ATAC-seq and CUT&Tag). Uncropped and unedited Western Blots are shown in Supplementary Figures 6 and 7 and source data are provided in Supplementary Data 1-3.
