# Supplementary material for: HNF4A guides the MLL4 complex to establish and maintain H3K4me1 at gene regulatory elements
Source: Commun Biol. 2024 Jan 31;7:144. doi: 10.1038/s42003-024-05835-0 (PMC10830483; doi:10.1038/s42003-024-05835-0)
Supplement: Supplementary file 1 — Supplementary Information [file 42003_2024_5835_MOESM1_ESM.pdf]

SUPPLEMENTARY INFORMATION

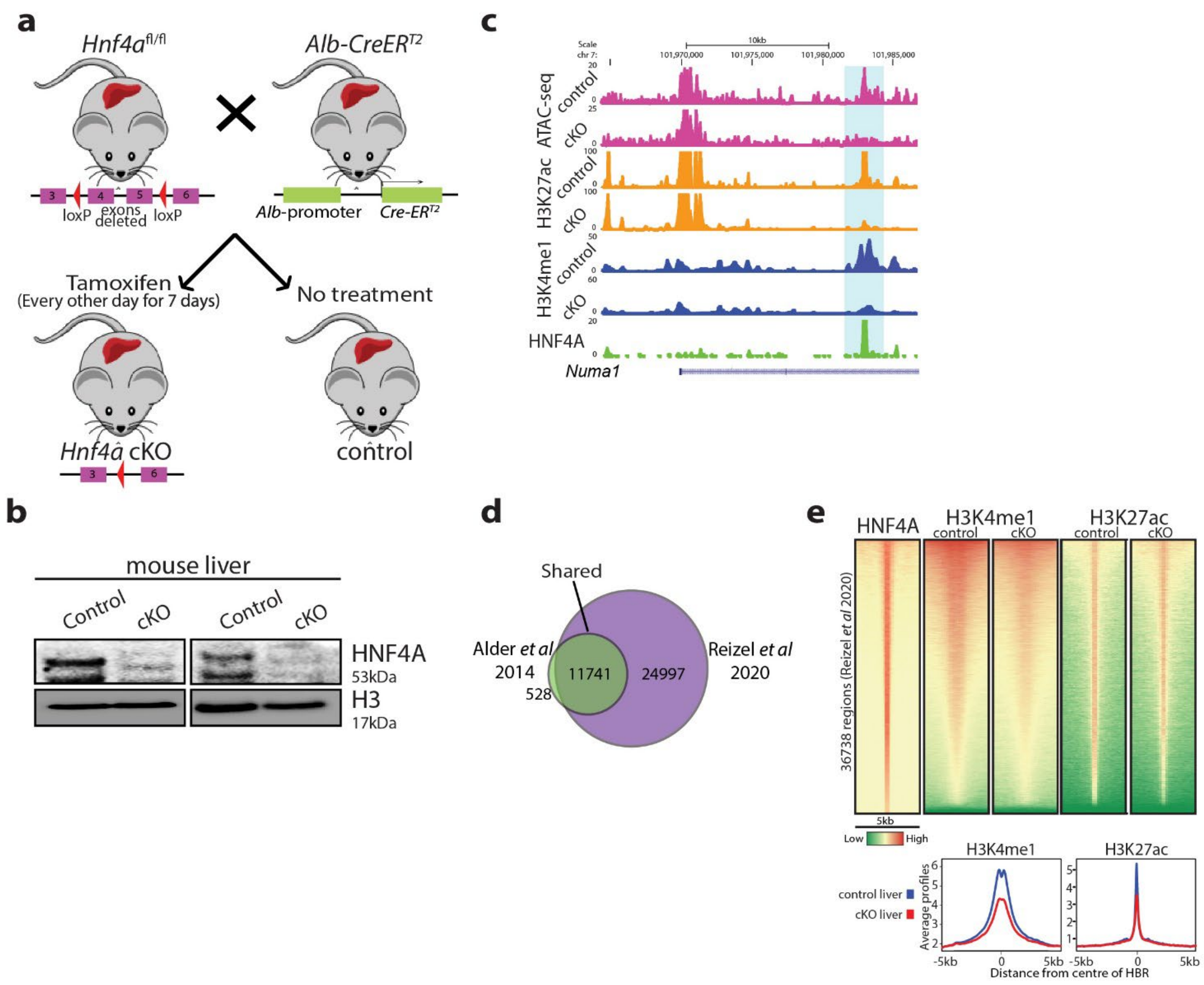

Supplementary Figure 1. Hnf4a cKO livers show reduced histone modifications at HNF4A-bound regions. *See also Fig. 1.*

a Depiction of how HNF4A was conditionally knocked out in the adult liver. All conditional knockout (cKO) livers used for experiments were following 7 days of tamoxifen treatment. Control animals were of the same genotype but received no tamoxifen. **b** Western blots showing loss of HNF4A in cKO compared to control with total H3 as loading control. Biological replicates are shown. **c** Genome browser of

Numa1 as an example of changes observed in cKO. HBR highlighted in blue. **d** Venn diagram comparing our HNF4A mouse adult liver dataset (Alder *et al* 2014) with HNF4A mouse adult liver dataset (Reizel *et al* 2020). **e** Heatmaps of our control and cKO data on the expanded Reizel dataset. Average profiles shown at the bottom illustrating the decrease in H3K4me1 and H3K27ac at HBRs in the expanded dataset.

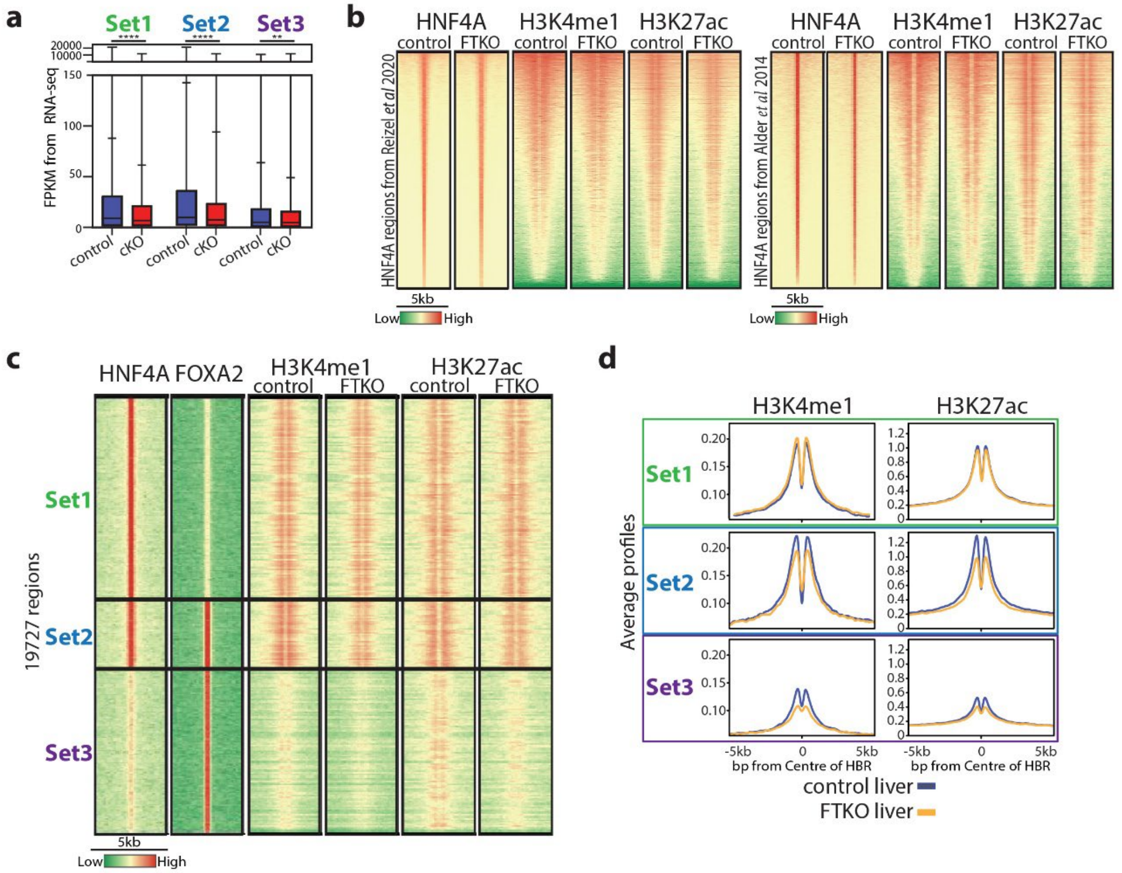

Supplementary Figure 2. HNF4A binding and its maintenance of histone modifications are unaffected by loss of all FOXA proteins. *See also Fig. 2.*

a Expression from RNA-seq for control vs cKO of genes associated with HBRs identified in each set. Line on bar graph designates median while line on whiskers designates mean. (****p-value<0.0001, **p-value<0.01). n=3 biologically independent samples. **b** Heatmaps showing HNF4A, H3K4me1 and H3K27ac in control and FOXA triple knockout (FTKO) across both the Reizel *et al* dataset and our dataset (HNF4A in mouse adult liver) around HBRs. **c** Heatmaps showing H3K4me1 and H3K27ac in control and FTKO from Reizel data across Set1 (HNF4A bound only), Set2 (shared regions where HNF4A and FOXA2 bind) and Set3 (FOXA2 bound only) around TF

bound regions. **d** Average profiles for H3K4me1 and H3K27ac for control and FTKO livers over Set1-Set3.

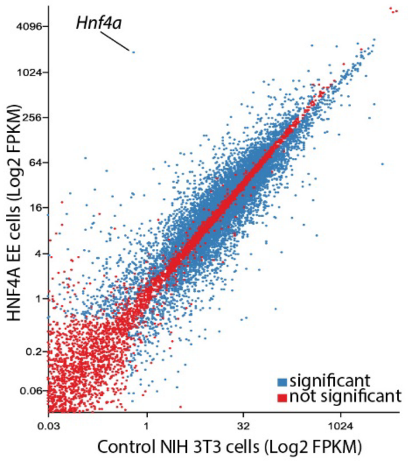

Supplementary Figure 3. HNF4A is highly expressed in cells ectopically expressing the mRNA linked with GFP. *See also Fig. 3.*

Scatterplot of RNA-seq for NIH 3T3 control (empty vector transduction) and HNF4A ectopic expression (EE; transduced with HNF4A expression plasmid). *Hnf4a* is indicated. Significantly differentially expressed genes are shown in blue.

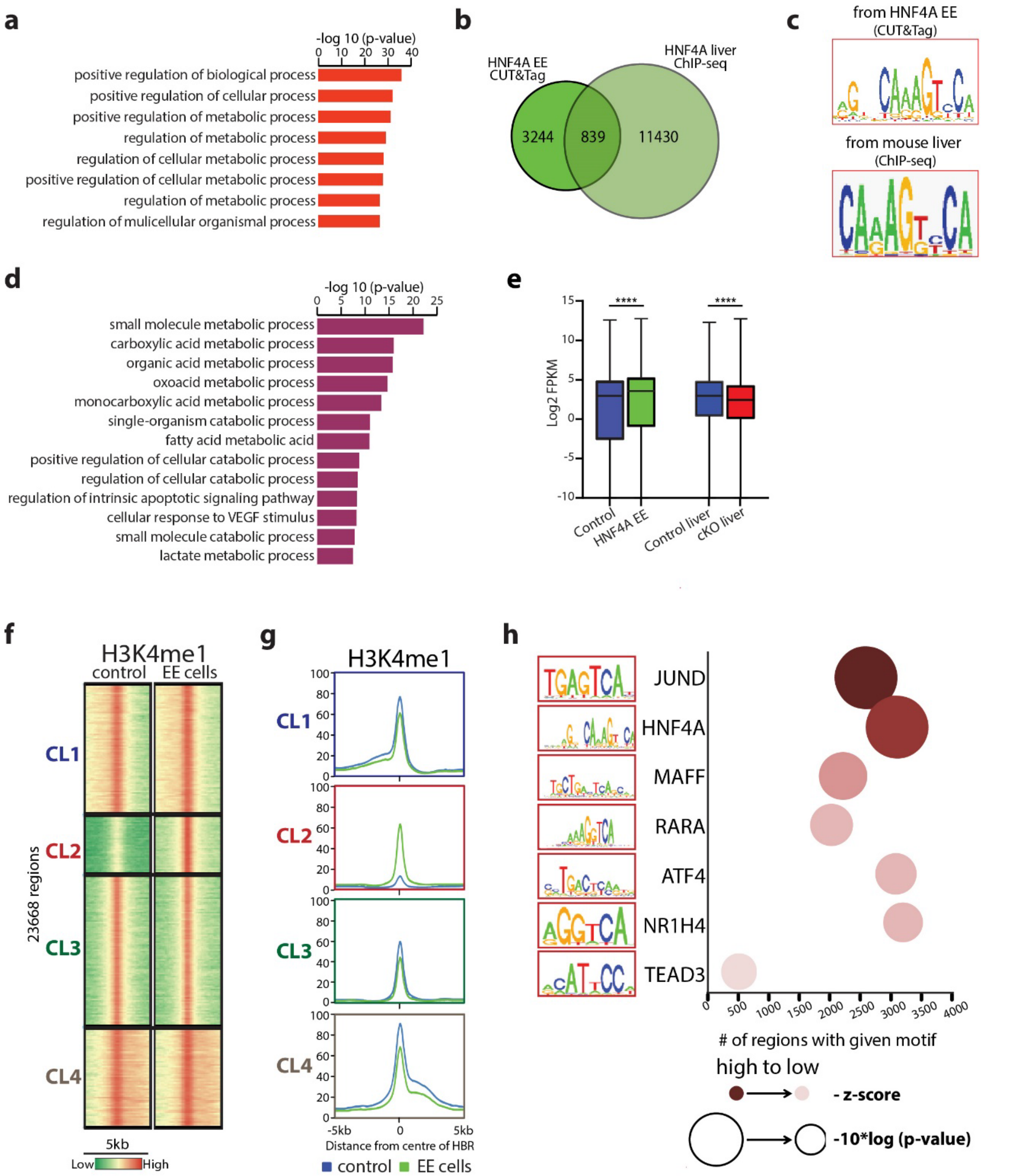

Supplementary Figure 4. HNF4A bound regions vary greatly based on cell type but HNF4A enhances H3K4me1 levels in either setting. *See also Fig. 4.*

a Gene association of 4083 HNF4A bound regions (HBRs) from 3T3 EE cells found metabolic processes enriched by Gene Ontology. Significance shown as –log 10 (p-

value). **b** Venn diagram showing overlap of HBRs (839 regions) between HBRs from HNF4A EE 3T3 cells and mouse adult liver. **c** HNF4A motifs found from CUT&Tag in HNF4A EE cells and ChIP-seq in adult mouse liver. **d** Gene Ontology analysis following gene association for the 839 shared regions between the two HNF4A datasets (3T3 cells and mouse adult liver) show that these genes are enriched for metabolic processes. **e** Expression analysis based on RNA-seq for the genes associated with the 839 regions in control compared to HNF4A EE and the control livers compared to cKO livers. Gene expression changes are statistically significant (****p-value<0.0001). n=3 biologically independent samples. **f** Heatmaps show that CL2 are gaining H3K4me1 when HNF4A is ectopically expressed All H3K4me1 regions found in EE cells are shown on the heatmaps. **g** Average profiles of H3K4me1 for control and EE cells for each cluster. **h** Motif analysis for CL2 demonstrating significance based on z-score (colour), p-value (size of circle) and number of regions with the given motif.

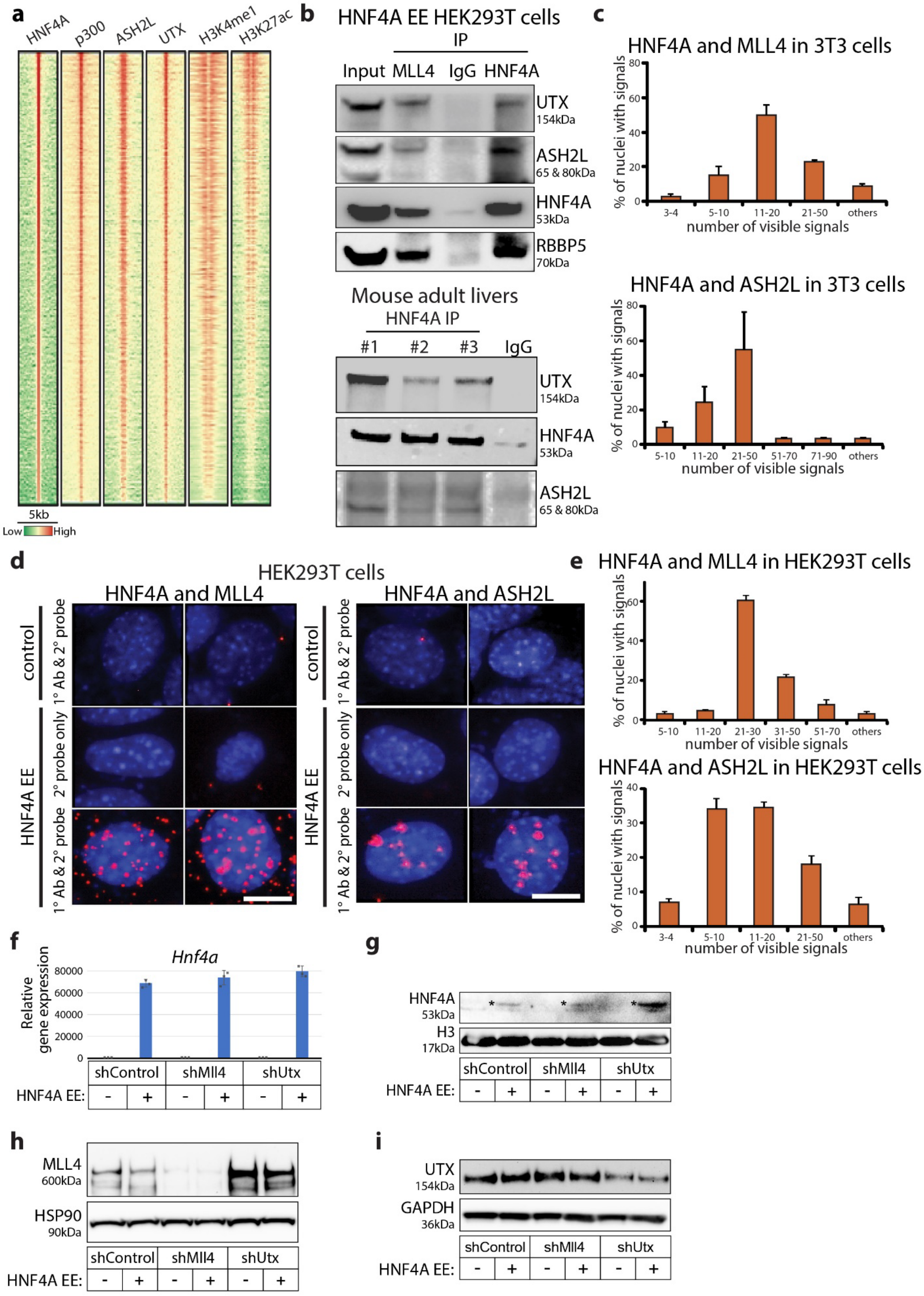

a
HNF4A
p300
ASH2L
UTX
H3K4me1
H3K27ac
5kb
Low
High
b
HNF4A EE HEK293T cells
IP
Input
MLL4
IgG
HNF4A
UTX
154kDa
ASH2L
65 & 80kDa
HNF4A
53kDa
RBBP5
70kDa
Mouse adult livers
HNF4A IP
#1
#2
#3
IgG
UTX
154kDa
HNF4A
53kDa
ASH2L
65 & 80kDa
c
HNF4A and MLL4 in 3T3 cells
% of nuclei with signals
number of visible signals
3-4
5-10
11-20
21-50
others
HNF4A and ASH2L in 3T3 cells
% of nuclei with signals
number of visible signals
5-10
11-20
21-50
51-70
71-90
others
d
HEK293T cells
HNF4A and MLL4
HNF4A and ASH2L
control
1° Ab & 2° probe
HNF4A EE
2° probe only
1° Ab & 2° probe
e
HNF4A and MLL4 in HEK293T cells
% of nuclei with signals
number of visible signals
5-10
11-20
21-30
31-50
51-70
others
HNF4A and ASH2L in HEK293T cells
% of nuclei with signals
number of visible signals
3-4
5-10
11-20
21-50
others
f
Hnf4a
Relative gene expression
80000
60000
40000
20000
0
shControl
shMll4
shUtx
HNF4A EE:
g
HNF4A
53kDa
H3
17kDa
shControl
shMll4
shUtx
HNF4A EE:
h
MLL4
600kDa
HSP90
90kDa
shControl
shMll4
shUtx
HNF4A EE:
i
UTX
154kDa
GAPDH
36kDa
shControl
shMll4
shUtx
HNF4A EE:

Supplementary Figure 5. HNF4A interacts with MLL4 complex components to direct H3K4me1 establishment. *See also Fig. 5.*

a Heatmaps around HNF4A bound regions identified in HepG2 cells show binding of p300, ASH2L, and UTX at HBRs. **b** Immunoprecipitation using MLL4 or HNF4A antibodies followed by western blotting with UTX, ASH2L, HNF4A or RBBP5 primary antibodies in HEK293T cells ectopically expressing HNF4A (top) or mouse adult liver (bottom; where HNF4A is endogenously expressed) show interactions are occurring between components of MLL4 complex and HNF4A. **c** Bar chart with standard deviation showing the percentage of nuclei with positive PLA for HNF4A and MLL4 (top) or HNF4A and ASH2L (bottom) signals in HNF4A EE 3T3 cells. Y-axis groups cells based on the number of detected interactions per cell. **d** PLA for HNF4A and MLL4 (left) or HNF4A and ASH2L (right) in HEK293T cells ectopically expressing HNF4A. Top two panels in each show negative controls. Red dots show positive signal in the bottom panel. Scale bars, 10 μm. **e** Bar charts with standard deviation plotting percentage of nuclei with specific number of visible signals in HEK293T cells. **f** The RT-qPCR shows increased *Hnf4a* expression after 72 hours from initial transduction. Error bars represent standard deviation. **g, h, i** Western blots show protein expression of HNF4A (**g**), MLL4 (**h**), and UTX (**i**). H3, HSP90 and GAPDH are used as loading controls. Asterisks (*) indicate the protein bands for HNF4A. n=3 field of views (**c, e**) or 3 technical replicates (**f**).

a

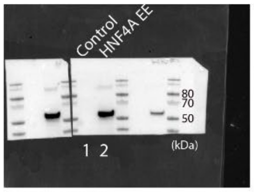

anti-HNF4A immunostaining
with pre-stained protein ladders

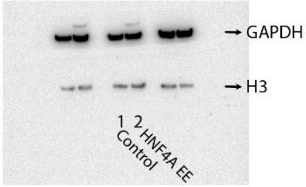

anti-GAPDH and anti-H3
immunostaining

b

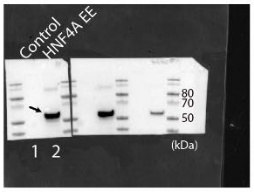

anti-HNF4A immunostaining
with pre-stained protein ladders

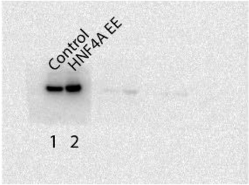

anti-H3K4me1 immunostaining

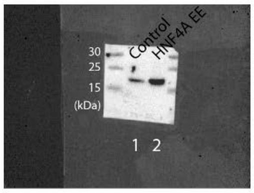

anti-H3K27ac immunostaining

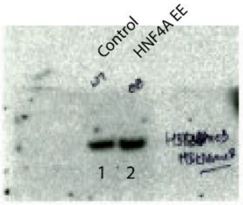

anti-H3K36me3 immunostaining

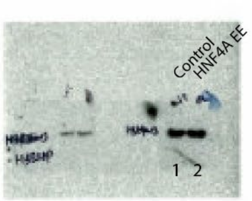

anti-H3K9me3 immunostaining

c

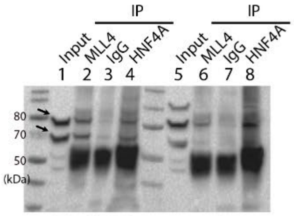

anti-ASH2L immunostaining
with pre-stained protein ladders

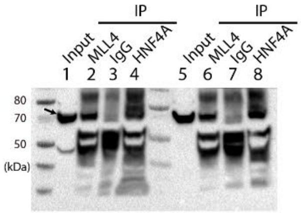

anti-RBBP5 immunostaining
with pre-stained protein ladders

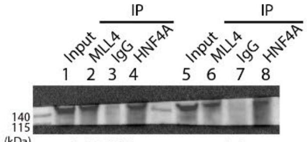

anti-UTX immunostaining
with pre-stained protein ladders

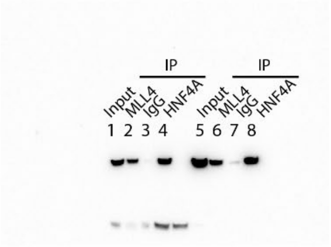

anti-HNF4A immunostaining

Supplementary Figure 6. Uncropped western blot images. *See also Figs. 3, 4, 5 and Supplementary Fig. 5.*

a, b Lanes 1 and 2 were used in Figs. 3c and 4b, respectively. **c** Fig. 5d employed lanes 1-4, while lanes 5-8 were utilized for Supplementary Fig. 5b. PageRuler™ Prestained Protein Ladder (Thermo), size marker.

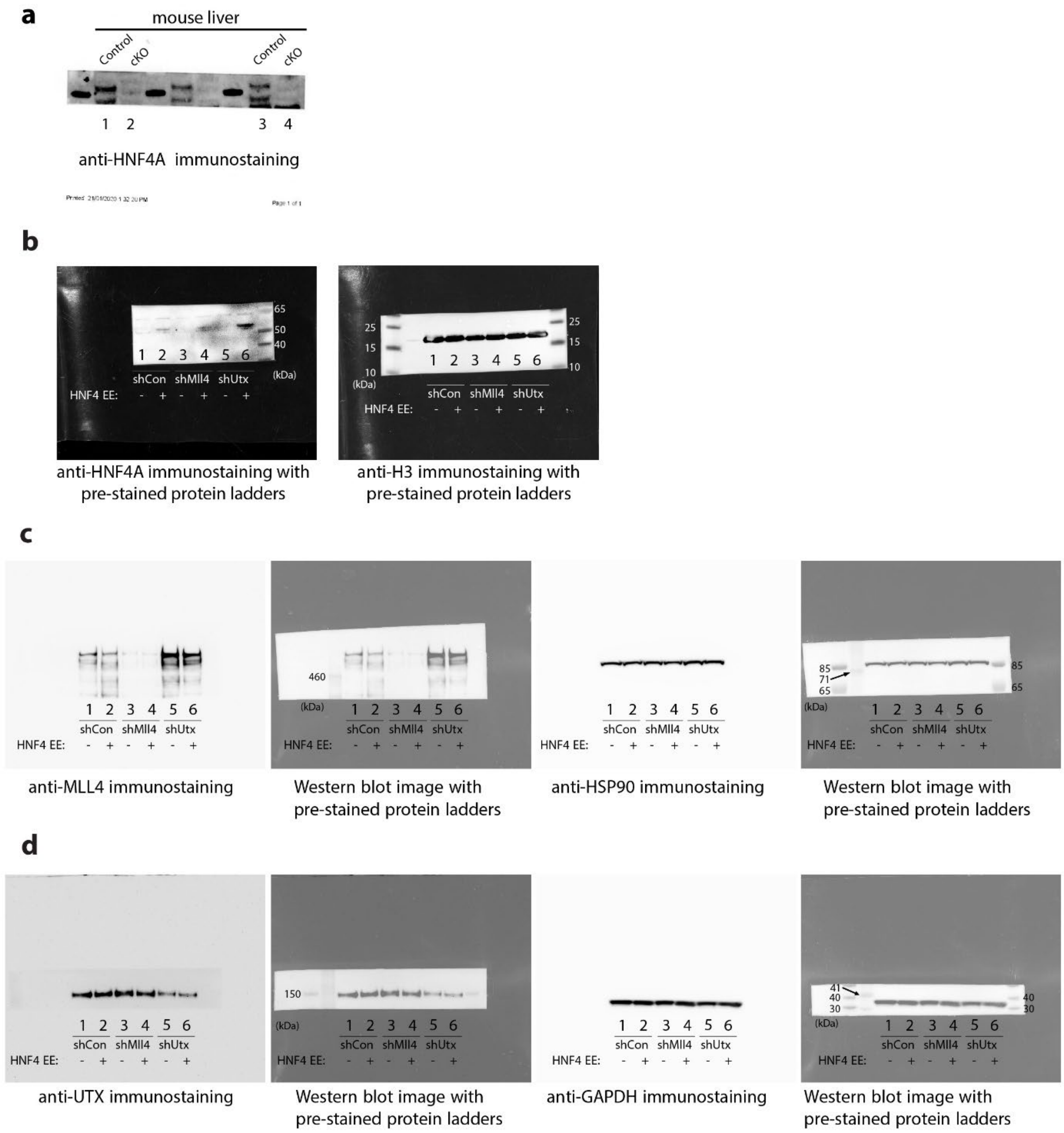

Supplementary Figure 7. Additional details and usage of western blot images. *See also Supplementary Figs. 1 and 5.*

a Western blot images of lanes 1-4 were used for Supplementary Fig. 1b. **b, c, d** Uncropped images were used for Supplementary Fig. 5g (**b**), Supplementary Fig. 5h

(**c**), and Supplementary Fig. 5i (**d**). HiMark™ Pre-stained Protein Standard (Invitrogen) and PageRuler™ Prestained Protein Ladder (Thermo) were used as size markers.

Supplementary Table 1. Materials used in this study

Primers used for q-RT PCR

Gene	Species	Forward sequence	Reverse sequence
Hnf4a	Mouse	AGCGTGAGGAAGAACCACAT	AGCCCGGAAGCACTTCTTA
Gapdh	Mouse	AGGTCGGTGTGAACGGATTTG	TGTAGACCATGTAGTTGAGGTCA
B-Actin	Mouse	GGCTGTATTCCCCTCCATCG	CCAGTTGGTAACAATGCCATGT
Apoc2	Mouse	TTCCCAACATCAGGATGACCA	TACTGGAGTGAGCCAGGATAG
Aqp1	Mouse	CTGCTGGCGATTGACTACA	AAATCCAGTGGTTTGAGAAGTTG

ChIP-qPCR Primers

Nnmt	Mouse	ACCCTCATTACATGGCTTGTGACC	GCAGTGGGCAGAACATCCAAAGAA
Prlr	Mouse	TCCCTTCATCTCGTCGGTGTTTGA	GCGGTATCATTGCTGTGTGGCATT
Rfx4	Mouse	ACAGGAGCCAGGAAAGGAACAGAA	AGGCATGCTTTGTTTAGCCTGAGC
Ido2	Mouse	CGAGGAGCAAAGTTCCATAGAGACAC	TTGGGAAAGAACAGTGGGTTTGGC
Agxt	Mouse	ATCAGCAAGTGAAGGCCACGAAAC	TGCAACTTGAGAAGCTGGCCATTG
Six1	Mouse	CTATCTTTGTGCCGGGTGTT	GGTCCCATTCTCCCTTGTTA
Apoc2	Mouse	TGAACTCCTGATCGTCCTGC	GGTGCTCAGGGTTCTAGGTT
Aqp1	Mouse	CTGCGGATAAGGCACAGAAA	GTACAGATAACCCAGAGGCAAG
Negative control	Mouse	TGGCTTGATGCCTATCCTCTGCAA	TGATCAGAACCAAGCAGAGGCAGT

Antibody used for ChiP-qpcr, ChIP-seq, CUT&TAG, CO-IP, PLA & Western Blot

Antibody	Company	Catalogue	Species	Application
H3K27ac	Abcam	ab4729	Rabbit	ChIP-seq and Western blot
H3K4me1	Diagenode	C154100037-50	Rabbit	ChIP-seq and Western blot
H3k4me3	Cell Signaling	9751	Rabbit	Western blot, ChIP-seq and PLA
H3K36me3	Abcam	ab9050	Rabbit	Western blot
H3K9me3	Abcam	ab8898	Rabbit	Western blot
H3	Active Motif	39064	Mouse	Western blot
MLL4	Millipore	ABE1867	Rabbit	CUT&TAG, Western blot & CO-IP
UTX	Cell Signaling	D3Q1I	Rabbit	Western blot
UTX	Abcam	ab154126	Rabbit	Western blot
ASH2	Bethyl Laboratories	A300-112A	Rabbit	Western blot & PLA
RBBP5	Bethyl Laboratories	A300-109A	Rabbit	Western blot
HNF4A	Santa Cruz Biotechnology	sc-8987	Rabbit	CUT&TAG, CO-IP, PLA, Western blot
HNF4A	Santa Cruz Biotechnology	sc-6556	Goat	CUT&TAG, CO-IP, PLA, Western blot
Normal Goat IgG	Santa Cruz Biotechnology	sc-2028	Goat	CUT&TAG, CO-IP, PLA, Western blot
Normal Rabbit IgG	Santa Cruz Biotechnology	sc-2027	Rabbit	CUT&TAG, CO-IP, PLA, Western blot
GAPDH	Invitrogen	AM4300	Mouse	Western blot
HSP90	Santa Cruz Biotechnology	sc-13119	Mouse	Western blot

Probes used for PLA

Probes name	Company	Catalogue
Anti Rabbit minus	Sigma/Duolink	DUO92005
Anti Goat Plus	Sigma/Duolink	DUO92003

mouse TRC shRNA

Gene	TRC ID	Gene ID	Species
Utx/Kdm6a	TRCN0000305239	22289	Mouse
Mll4/Kmt2d	TRCN0000239234	381022	Mouse
