# Supplementary material for: HNF4A guides the MLL4 complex to establish and maintain H3K4me1 at gene regulatory elements
Source: Commun Biol. 2024 Jan 31;7:144. doi: 10.1038/s42003-024-05835-0 (PMC10830483; doi:10.1038/s42003-024-05835-0)
Supplement: Supplementary file 2 — Description of additional supplementary files [file 42003_2024_5835_MOESM2_ESM.docx]

Description of Additional Supplementary Files

**File name:** Supplementary Data 1

**Description:** RNA-seq of control and HNF4A cKO livers

**File name:** Supplementary Data 2

**Description:** RNA-seq in control NIH 3T3 cells and HNF4A ectopically expressing NIH 3T3 cells

**File name:** Supplementary Data 3

**Description:** Numerical source data for graphs
