# Supplementary material for: HNF4A guides the MLL4 complex to establish and maintain H3K4me1 at gene regulatory elements
Source: Commun Biol. 2024 Jan 31;7:144. doi: 10.1038/s42003-024-05835-0 (PMC10830483; doi:10.1038/s42003-024-05835-0)
Supplement: Supplementary file 6 — Reporting Summary [file 42003_2024_5835_MOESM6_ESM.pdf]

nature portfolio

Corresponding author(s): Pamela Hoodless

Last updated by author(s): Jan 15, 2024

Reporting Summary

Nature Portfolio wishes to improve the reproducibility of the work that we publish. This form provides structure for consistency and transparency in reporting. For further information on Nature Portfolio policies, see our Editorial Policies and the Editorial Policy Checklist.

Statistics

For all statistical analyses, confirm that the following items are present in the figure legend, table legend, main text, or Methods section.

n/a	Confirmed	
☐	☒	The exact sample size (*n*) for each experimental group/condition, given as a discrete number and unit of measurement
☐	☒	A statement on whether measurements were taken from distinct samples or whether the same sample was measured repeatedly
☐	☒	The statistical test(s) used AND whether they are one- or two-sided<br>*Only common tests should be described solely by name; describe more complex techniques in the Methods section.*
☒	☐	A description of all covariates tested
☒	☐	A description of any assumptions or corrections, such as tests of normality and adjustment for multiple comparisons
☐	☒	A full description of the statistical parameters including central tendency (e.g. means) or other basic estimates (e.g. regression coefficient) AND variation (e.g. standard deviation) or associated estimates of uncertainty (e.g. confidence intervals)
☐	☒	For null hypothesis testing, the test statistic (e.g. *F*, *t*, *r*) with confidence intervals, effect sizes, degrees of freedom and *P* value noted<br>*Give P values as exact values whenever suitable.*
☒	☐	For Bayesian analysis, information on the choice of priors and Markov chain Monte Carlo settings
☒	☐	For hierarchical and complex designs, identification of the appropriate level for tests and full reporting of outcomes
☒	☐	Estimates of effect sizes (e.g. Cohen's *d*, Pearson's *r*), indicating how they were calculated

Our web collection on statistics for biologists contains articles on many of the points above.

Software and code

Policy information about availability of computer code

Data collection	*Provide a description of all commercial, open source and custom code used to collect the data in this study, specifying the version used OR state that no software was used.*
Data analysis	*Provide a description of all commercial, open source and custom code used to analyse the data in this study, specifying the version used OR state that no software was used.*

For manuscripts utilizing custom algorithms or software that are central to the research but not yet described in published literature, software must be made available to editors and reviewers. We strongly encourage code deposition in a community repository (e.g. GitHub). See the Nature Portfolio guidelines for submitting code & software for further information.

Data

Policy information about availability of data

All manuscripts must include a data availability statement. This statement should provide the following information, where applicable:

- Accession codes, unique identifiers, or web links for publicly available datasets
- A description of any restrictions on data availability
- For clinical datasets or third party data, please ensure that the statement adheres to our policy

The data generated in this manuscript is available through GEO accession numbers: GSE210842 (RNA-seq) and GSE211123 (ATAC-seq and CUT&Tag).

Research involving human participants, their data, or biological material

Policy information about studies with human participants or human data. See also policy information about sex, gender (identity/presentation), and sexual orientation and race, ethnicity and racism.

Reporting on sex and gender	N/A
Reporting on race, ethnicity, or other socially relevant groupings	N/A
Population characteristics	N/A
Recruitment	N/A
Ethics oversight	N/A

Note that full information on the approval of the study protocol must also be provided in the manuscript.

Field-specific reporting

Please select the one below that is the best fit for your research. If you are not sure, read the appropriate sections before making your selection.

☒ Life sciences ☐ Behavioural & social sciences ☐ Ecological, evolutionary & environmental sciences

For a reference copy of the document with all sections, see nature.com/documents/nr-reporting-summary-flat.pdf

Life sciences study design

All studies must disclose on these points even when the disclosure is negative.

Sample size	Sample size varied based on experiment as described.
Data exclusions	No data was excluded
Replication	Biological replicates were performs as described in Materials and Methods and in Figure Legends.
Randomization	Randomization was not performed in this study.
Blinding	No blinding was used in this study.

Reporting for specific materials, systems and methods

We require information from authors about some types of materials, experimental systems and methods used in many studies. Here, indicate whether each material, system or method listed is relevant to your study. If you are not sure if a list item applies to your research, read the appropriate section before selecting a response.

Materials & experimental systems

n/a	Involved in the study	
☐	☒	Antibodies
☐	☒	Eukaryotic cell lines
☒	☐	Palaeontology and archaeology
☐	☒	Animals and other organisms
☒	☐	Clinical data
☒	☐	Dual use research of concern
☒	☐	Plants

Methods

n/a	Involved in the study	
☐	☒	ChIP-seq
☒	☐	Flow cytometry
☒	☐	MRI-based neuroimaging

Antibodies

Antibodies used	See Supplementary Table 1
Validation	All antibodies were commercial and were tested using western blotting.

Eukaryotic cell lines

Policy information about cell lines and Sex and Gender in Research

Cell line source(s)	NIH 3T3, HEK293T and HepG2 were purchased from American Type Culture Collection (ATCC)
Authentication	All cell lines used in this study were authenticated by ATCC
Mycoplasma contamination	Cell lines were tested for mycoplasma contamination, when required.
Commonly misidentified lines (See ICLAC register)	*Name any commonly misidentified cell lines used in the study and provide a rationale for their use.*

Animals and other research organisms

Policy information about studies involving animals; ARRIVE guidelines recommended for reporting animal research, and Sex and Gender in Research

Laboratory animals	For most experiments, 8-week-old female mice (C57BL6/J) were used. For Hnf4a cKO mice, mice with a tamoxifen inducible, Albumin promoter-driven Cre (Alb-CreERT2) were crossed with Hnf4afl/fl mice.
Wild animals	N/A
Reporting on sex	Female
Field-collected samples	N/A
Ethics oversight	Animal work was approved by the Animal Care Committee (University of British Columbia) and National Cancer Institute Animal Care and Use Committee

Note that full information on the approval of the study protocol must also be provided in the manuscript.

Plants

Seed stocks	*Report on the source of all seed stocks or other plant material used. If applicable, state the seed stock centre and catalogue number. If plant specimens were collected from the field, describe the collection location, date and sampling procedures.*
Novel plant genotypes	*Describe the methods by which all novel plant genotypes were produced. This includes those generated by transgenic approaches, gene editing, chemical/radiation-based mutagenesis and hybridization. For transgenic lines, describe the transformation method, the number of independent lines analyzed and the generation upon which experiments were performed. For gene-edited lines, describe the editor used, the endogenous sequence targeted for editing, the targeting guide RNA sequence (if applicable) and how the editor was applied.*
Authentication	*Describe any authentication procedures for each seed stock used or novel genotype generated. Describe any experiments used to assess the effect of a mutation and, where applicable, how potential secondary effects (e.g. second site T-DNA insertions, mosiacism, off-target gene editing) were examined.*

ChIP-seq

Data deposition

☒ Confirm that both raw and final processed data have been deposited in a public database such as GEO.

☒ Confirm that you have deposited or provided access to graph files (e.g. BED files) for the called peaks.

Data access links *May remain private before publication.*	https://www.ncbi.nlm.nih.gov/geo/query/acc.cgi?acc=GSE211123
Files in database submission	1-WT-liv1-H3K4me1-Jan-22-2020_S7_L001_R1_001.fastq.gz<br>1-WT-liv1-H3K4me1-Jan-22-2020_S7_L002_R1_001.fastq.gz<br>1-WT-liv1-H3K4me1-Jan-22-2020_S7_L003_R1_001.fastq.gz<br>1-WT-liv1-H3K4me1-Jan-22-2020_S7_L004_R1_001.fastq.gz<br>1-WT-liv1-H3K4me1-Jan-22-2020_S21_L001_R1_001.fastq.gz<br>1-WT-liv1-H3K4me1-Jan-22-2020_S21_L002_R1_001.fastq.gz<br>1-WT-liv1-H3K4me1-Jan-22-2020_S21_L003_R1_001.fastq.gz<br>1-WT-liv1-H3K4me1-Jan-22-2020_S21_L004_R1_001.fastq.gz<br>8-KO-liv5-H3K4me1-Jan-22-2020_S8_L001_R1_001.fastq.gz<br>8-KO-liv5-H3K4me1-Jan-22-2020_S8_L002_R1_001.fastq.gz<br>8-KO-liv5-H3K4me1-Jan-22-2020_S8_L003_R1_001.fastq.gz<br>8-KO-liv5-H3K4me1-Jan-22-2020_S8_L004_R1_001.fastq.gz<br>8-KO-liv5-H3K4me1-Jan-22-2020_S22_L001_R1_001.fastq.gz

8-KO-liv5-H3K4me1-Jan-22-2020_S22_L002_R1_001.fastq.gz
8-KO-liv5-H3K4me1-Jan-22-2020_S22_L003_R1_001.fastq.gz
8-KO-liv5-H3K4me1-Jan-22-2020_S22_L004_R1_001.fastq.gz
WTliver-ATAC_S3_L001_R1_001.fastq.gz
WTliver-ATAC_S3_L002_R1_001.fastq.gz
WTliver-ATAC_S3_L003_R1_001.fastq.gz
WTliver-ATAC_S3_L004_R1_001.fastq.gz
KOliver-ATAC_S4_L001_R1_001.fastq.gz
KOliver-ATAC_S4_L002_R1_001.fastq.gz
KOliver-ATAC_S4_L003_R1_001.fastq.gz
KOliver-ATAC_S4_L004_R1_001.fastq.gz
C-T-WTliver-H3K27ac_S13_L001_R1_001.fastq.gz
C-T-WTliver-H3K27ac_S13_L002_R1_001.fastq.gz
C-T-WTliver-H3K27ac_S13_L003_R1_001.fastq.gz
C-T-WTliver-H3K27ac_S13_L004_R1_001.fastq.gz
C-T-KO-liver-H3K27ac_S14_L001_R1_001.fastq.gz
C-T-KO-liver-H3K27ac_S14_L002_R1_001.fastq.gz
C-T-KO-liver-H3K27ac_S14_L003_R1_001.fastq.gz
C-T-KO-liver-H3K27ac_S14_L004_R1_001.fastq.gz
C-T-WT-liver-MLL4_S17_L001_R1_001.fastq.gz
C-T-WT-liver-MLL4_S17_L002_R1_001.fastq.gz
C-T-WT-liver-MLL4_S17_L003_R1_001.fastq.gz
C-T-WT-liver-MLL4_S17_L004_R1_001.fastq.gz
C-T-KO-liver-MLL4_S18_L001_R1_001.fastq.gz
C-T-KO-liver-MLL4_S18_L002_R1_001.fastq.gz
C-T-KO-liver-MLL4_S18_L003_R1_001.fastq.gz
C-T-KO-liver-MLL4_S18_L004_R1_001.fastq.gz
Control-H3K4me1-2-5_S16_L001_R1_001.fastq.gz
Control-H3K4me1-2-5_S16_L002_R1_001.fastq.gz
Control-H3K4me1-2-5_S16_L003_R1_001.fastq.gz
Control-H3K4me1-2-5_S16_L004_R1_001.fastq.gz
HNF4A-OE-H3K4me1-2-6_S18_L001_R1_001.fastq.gz
HNF4A-OE-H3K4me1-2-6_S18_L002_R1_001.fastq.gz
HNF4A-OE-H3K4me1-2-6_S18_L003_R1_001.fastq.gz
HNF4A-OE-H3K4me1-2-6_S18_L004_R1_001.fastq.gz
C-T-C-3T3-H3K27ac_S8_L001_R1_001.fastq.gz
C-T-C-3T3-H3K27ac_S8_L002_R1_001.fastq.gz
C-T-C-3T3-H3K27ac_S8_L003_R1_001.fastq.gz
C-T-C-3T3-H3K27ac_S8_L004_R1_001.fastq.gz
C-T-OE3T3-H3K27ac_S9_L001_R1_001.fastq.gz
C-T-OE3T3-H3K27ac_S9_L002_R1_001.fastq.gz
C-T-OE3T3-H3K27ac_S9_L003_R1_001.fastq.gz
C-T-OE3T3-H3K27ac_S9_L004_R1_001.fastq.gz
C-T-C3T3-MLL4_S9_L001_R1_001.fastq.gz
C-T-C3T3-MLL4_S9_L002_R1_001.fastq.gz
C-T-C3T3-MLL4_S9_L003_R1_001.fastq.gz
C-T-C3T3-MLL4_S9_L004_R1_001.fastq.gz
C-T-OE3T3-MLL4_S10_L001_R1_001.fastq.gz
C-T-OE3T3-MLL4_S10_L002_R1_001.fastq.gz
C-T-OE3T3-MLL4_S10_L003_R1_001.fastq.gz
C-T-OE3T3-MLL4_S10_L004_R1_001.fastq.gz
3T3-ATAC-ctrl_S20_L001_R1_001.fastq.gz
3T3-ATAC-ctrl_S20_L002_R1_001.fastq.gz
3T3-ATAC-ctrl_S20_L003_R1_001.fastq.gz
3T3-ATAC-ctrl_S20_L004_R1_001.fastq.gz
3T3-ATAC-HNF4AOE_S19_L001_R1_001.fastq.gz
3T3-ATAC-HNF4AOE_S19_L002_R1_001.fastq.gz
3T3-ATAC-HNF4AOE_S19_L003_R1_001.fastq.gz
3T3-ATAC-HNF4AOE_S19_L004_R1_001.fastq.gz

Genome browser session
(e.g. UCSC)

N/A

Methodology

Replicates

Chip-seq, CUT&Tag, and ATAC-seq all only had a single replicate. RNA-seq samples were done in triplicate (biological replicates).

Sequencing depth

WT-liver-H3K4me1-ChIP 29,127,167
KO-liver-H3K4me1-ChIP 64,449,569
WT-liver-ATAC 19,370,282
KO-liver-ATAC 17,985,328
WT-liver-H3K27ac-CT 6,230,541
KO-liver-H3K27ac-CT 8,089,727
WT-liver-MLL4-CT 3,665,739
KO-liver-MLL4-CT 4,198,815

3T3 HNF4a-CT 5,770,512
3T3 Control-H3K4me1-CT 8,066,322
3T3 HNF4OE-H3K4me1-CT 13,688,62
3T3 Control-H3K27ac-CT 7,293,180
3T3 HNF4OE-H3K27ac-CT 6,174,401
3T3 Control-MLL4-CT 7,073,950
3T3 HNF4OE-MLL4-CT 5,996,203
3T3 Control-ATAC 19,767,488
3T3 HNF4OE-ATAC 15,552,397

Antibodies

H3K27ac (Abcam, ab4729)
H3K4me1 (Diagenode, C154100037-50)
MLL4 (Millipore, ABE1867)
HNF4A (Santa Cruz, sc-8987)

Peak calling parameters

MACS2 was used to call peaks using the parameters "-p 0.01 -f BAMPE -g hs" for the CUT&RUN and CUT&Tag data, and "-p 0.01 -g hs --nomodel --shift -75 --extsize 150" for the ATAC-seq data.

Data quality

Quality was assessed by FastQC.
All reads were mapped to the mm10 genome using the BWA-MEM aligner version 1.1.4
Duplicate reads were marked by Picard v2.1.1
BAM files were filtered using the encode_task_filter.py script from the ENCODE chip-seq-pipeline2.
MACS2 was used to call peaks using the parameters "-p 0.01 -f BAMPE -g hs" for the CUT&RUN and CUT&Tag data, and "-p 0.01 -g hs --nomodel --shift -75 --extsize 150" for the ATAC-seq data.
All bed and bw files were filtered using the ENCODE blacklist.

Software

FastQC
BWA-MEM aligner 1.1.4
Picard 2.1.1
MACS2
SAMtools
MACS2 bdgcmp (using fold enrichment option for ATAC-seq data)
Bedtools
deepTools
Cistrome (SitePro for profile plots)
Galaxy
IGV
Motif analysis: Seqpos
ChAsE
GREAT (gene associations)
